# Genome-wide association analyses for carcass quality in crossbred beef cattle

**DOI:** 10.1186/1471-2156-14-80

**Published:** 2013-09-11

**Authors:** Duc Lu, Mehdi Sargolzaei, Matthew Kelly, Gordon Vander Voort, Zhiquan Wang, Ira Mandell, Stephen Moore, Graham Plastow, Stephen Paul Miller

**Affiliations:** 1 Centre for Genetic Improvement of Livestock, Department of Animal and Poultry Science, University of Guelph, 50 Stone Road East, Guelph, Ontario N1G 2W1, Canada; 2 Livestock Gentec, Department of Agricultural, Food and Nutritional Science, University of Alberta, 1400 College Plaza, 8215 112 Street, Edmonton, Alberta T6G 2C8, Canada; 3 L’Alliance Boviteq, 19320 Grand rang Saint-François, Saint-Hyacinthe, Quebec J2T 5H1, Canada; 4 Centre for Animal Science, Queensland Alliance for Agriculture & Food Innovation, University of Queensland, St Lucia, Queensland 4072, Australia

**Keywords:** Single nucleotide polymorphism, Chromosome regions, Beef carcass quality

## Abstract

**Background:**

Genetic improvement of beef quality will benefit both producers and consumers, and can be achieved by selecting animals that carry desired quantitative trait nucleotides (QTN), which result from intensive searches using genetic markers. This paper presents a genome-wide association approach utilizing single nucleotide polymorphisms (SNP) in the Illumina BovineSNP50 BeadChip to seek genomic regions that potentially harbor genes or QTN underlying variation in carcass quality of beef cattle.

This study used 747 genotyped animals, mainly crossbred, with phenotypes on twelve carcass quality traits, including hot carcass weight (HCW), back fat thickness (BF), *Longissimus dorsi* muscle area or ribeye area (REA), marbling scores (MRB), lean yield grade by Beef Improvement Federation formulae (BIFYLD), steak tenderness by Warner-Bratzler shear force 7-day post-mortem (LM7D) as well as body composition as determined by partial rib (IMPS 103) dissection presented as a percentage of total rib weight including body cavity fat (BDFR), lean (LNR), bone (BNR), intermuscular fat (INFR), subcutaneous fat (SQFR), and total fat (TLFR).

**Results:**

At the genome wide level false discovery rate (FDR < 10%), eight SNP were found significantly associated with HCW. Seven of these SNP were located on *Bos taurus autosome* (BTA) 6. At a less stringent significance level (P < 0.001), 520 SNP were found significantly associated with mostly individual traits (473 SNP), and multiple traits (47 SNP). Of these significant SNP, 48 were located on BTA6, and 22 of them were in association with hot carcass weight. There were 53 SNP associated with percentage of rib bone, and 12 of them were on BTA20. The rest of the significant SNP were scattered over other chromosomes. They accounted for 1.90 - 5.89% of the phenotypic variance of the traits. A region of approximately 4 Mbp long on BTA6 was found to be a potential area to harbor candidate genes influencing growth. One marker on BTA25 accounting for 2.67% of the variation in LM7D may be worth further investigation for the improvement of beef tenderness.

**Conclusion:**

This study provides useful information to further assist the identification of chromosome regions and subsequently genes affecting carcass quality traits in beef cattle. It also revealed many SNP that acted pleiotropically to affect carcass quality. This knowledge is important in selecting subsets of SNP to improve the performance of beef cattle.

## Background

Beef quality and consumer satisfaction are important to the beef industry. Beef quality contributes to consumers’ decision to purchase beef. An improvement of beef quality can increase demand, benefiting both producers and consumers [1]. Leading beef quality defects and subsequent lost carcass value include insufficient marbling and low quality grades, lack of cattle uniformity, inadequate beef tenderness, high yield grades, and excessive carcass weight [2,3]. Standards for beef quality have been set out and carcasses not meeting the standards are discounted [2].

Efforts to improve beef quality have been made through conventional beef cattle breed improvement programs using pedigree and performance records to estimate expected progeny differences (EPD). Although Real-time ultrasound imaging has been used on live animals to measure a number of traits, such as *Longissimus dorsi* muscle area, and backfat, it evaluates marbling through ultrasound intramuscular fat, and cannot measure tenderness. These two traits, tenderness and marbling, can only be obtained after the animal has been slaughtered. Thus there exists a time-delay in identifying elite animals before they can be widely used, and is the major limitation to improving current rates of genetic improvement [4]. Selection programs that incorporate either subsets of markers on select regions of the genome, for instance markers for growth and feed efficiency, as well as carcass quality [5], or double-muscling DNA test [6], or a large number of markers covering the whole genome in genomic selection that has been reported to result in a 32% improvement in accuracy compared to parental average breeding values [7], have drawn the attention of both beef researchers and producers. In beef cattle, American Angus Association has started incorporating genetic marker information from a 50,000 marker panel into their weekly carcass EPD evaluation program [8], American Simmental Association has applied marker assisted EPD for beef tenderness [9], and North American Limousin Foundation announced its commencement of genomic-enhanced EPDs as of December 2012 [10].

As for multi-breed beef cattle, which constitute a large proportion of the beef population, genomic selection is still in the development stage. However, immediate applications of the 50K panel can include searching the genome for regions that may contain causative mutations underlying genetic variation of the carcass quality traits. The study reported here was designed for this purpose.

## Methods

### Animals and phenotypic data

Seven hundred and forty-seven animals were genotyped, using the Illumina BovineSNP50 BeadChip, including 713 males born between 1998 and 2006, and 34 feedlot heifers born between 1999 and 2005. These 747 animals consisted of 16 purebreds and 731 crossbreds. Number of animals with at least 50% of one of the six major breeds (Angus – AN, Charolais – CH, Simmental – SM, Piedmontese – PI, Limousin – LM, Gelbvieh – GV) are presented in Table 1. The rest of the animals, 97 individuals, were made of other breed combinations. The 747 animals were parented by 160 sires (maximum 17 progeny per sire) and 554 dams. The extended pedigree of these 747 animals contains 3,116 individuals, and the longest ancestral path was 11.

**Table 1:** Distribution of animals among six major breeds

**% of breed**	**AN**	**SM**	**CH**	**PI**	**LM**	**GV**
50-70	296	142	50	127	15	15
>75	17	2	0	0	0	0
100	1	0	3	12	0	0

*AN* Angus, *SM* Simmental, *CH* Charolais, *PI* Piedmontese, *LM* Limousin, *GV* Gelbvieh.

The test animals were born in one of the three cooperating herds: Elora Beef Research Centre (EBRC), New Liskeard Agriculture Research Station (NLARS), and Agriculture and Agri-food Canada Kapuskasing Experimental Farm (KAP). Cows at these three herds were bred to mostly purebred sires through the extensive use of artificial insemination. Calves were raised with their dams either on pasture or in group pens. NLARS and KAP cattle were transported to EBRC at weaning around 200 days of age to be fed under feedlot conditions typical of the Ontario beef industry.

The animals were slaughtered at an average age of 452 days, at a federally inspected abattoir operated by the University of Guelph. This facility does not use electrical stimulation, which can increase muscle tenderness. Hot carcass weight (HCW, kg) is the weight of carcass after harvest and removal of the head, the fore-shank below the knee joint, the hind-shank below the hock joint, gastrointestinal tracts and internal organs. Back fat thickness (BF, mm) was measured perpendicular to the outside surface at the 12^th^- and 13^th^-rib interface, three-fourths of the length of the *Longissimus* muscle from the backbone. Ribeye area (REA, sq.cm) was the measure of *Longissimus* muscle area at the 12^th^- and 13^th^-rib interface using a tracing of the muscle with the area quantified using an electronic planimeter (MOP-3; Carl Zeiss Canada LTD., Toronto, ON.). *Longissimus* muscle was assessed for subjective marbling score (MRB) from a scale of 1 (devoid marbling) to 10 (abundant marbling) based on the size and distribution of flecks of intramuscular fat in the *Longissimus* muscle at the 12^th^- and 13^th^-rib interface. Lean yield grade by Beef Improvement Federation formulae (BIFYLD) was calculated using the following formula Yield Grade = 2.50 + (2.5 × Adj. fat thickness, in.) + (0.2 × Kidney, pelvic, and heart fat, %) + (0.0038 × Hot carcass wt., lb.) – (0.32 × REA, sq. in.). Low BIFYLD values mean higher yields of retail product yield.

Steak tenderness was determined using Warner-Bratzler shear force to measure the amount of force (kg) required to cut through cooked *Longissimus dorsi* that had been aged for 7 days post-mortem (LM7D). Steaks were thawed for 48 hours at 1.5°C, trimmed of external fat and epimysium, and weighed prior to cooking. Steaks were cooked to an internal temperature of 70°C on a Garland Grill (ED-30B broiler, Garland Commercial Range Ltd., Mississauga, ON). Steak temperature was continually monitored by a Type K flexible high temperature thermocouple (Omega, Laval, Que) inserted into the geometric centre of each steak. Steaks were turned when they reached an internal temperature of 40°C. Cooked steaks were weighed, placed into individual bags, and immediately chilled in ice water to stop the cooking process. Steaks were then transferred to a chill cooler where they were stored at 1.5°C for 24 hours prior to coring. After equilibrating to room temperature, approximately eight cores of 1.5 cm were removed parallel to the muscle fibres from each steak using a drill press mounted corer. Cores were sheared using a Warner-Bratzler blade on a TA-XT Plus texture analyzer (Texture Technologies Corp., Scarsdale, NY) with crosshead speed set at 3.3 mm s^-1^. Peak shear force was determined using a custom macro program in Stable Microsystems Exponent software, with the average of the eight peak force values used in data analysis as the shear force value for each animal.

Carcass composition was assessed based on complete separation of a 21 cm rib section into body cavity fat (BDFR), lean (LNR), bone (BNR), intermuscular fat (INFR), subcutaneous fat (SQFR), while total fat (TLFR) was the sum of BDFR, INFR, and SQFR, following a modification of the procedure originally developed by Hankins and Howe [11]. The traits were expressed as proportions of the total rib weight.

### Genotypic data

DNA extracted either from blood or meat samples was genotyped using the Illumina BovineSNP50_v1 Beadchip at the Alberta Bovine Genomics laboratory. All 51,620 SNP in this panel went through quality control that excluded SNP with spurious position, low call rates (< 95%), parentage error, out of Hardy Weinberg equilibrium (P < 0.01) or less than 10% minor allele frequency (MAF). A total number of 38,745 SNP on 29 *Bos taurus autosomes* (BTA) remained for further analysis. The number of SNP varied among the chromosomes, with BTA1 having the highest number of SNP (2,082) and BTA28 having the fewest (608); the longest SNP interval was identified on BTA5 (1.53 Mbp). However average SNP intervals were relatively consistent among the chromosomes, and the overall average distance between two adjacent SNP was 70 kbp.

### Statistical analysis

Heritabilities and genetic correlations among the traits were estimated utilizing analytical gradients [12] in the REML VCE 6.0 package [13] with the model below excluding the allele substitution effect. To account for population stratification in association analysis, the animals were clustered into groups, using pair-wise population concordance test (PPC) at significance level of 0.05 from PLINK v1.07 [14]. A univariate animal model to estimate the allele substitution effect at each locus, *y*_*ijkmnpl*_ = + *γ*_*1*_(*age*_*ijkmnpl*_) + *γ*_*2*_(*h*_*ijkmnpl*_) *+ sex*_*i*_*+*$\sum_{j=1}^{6}\beta_{j}b_{j}$*+ t*_*k*_ *+ g*_*m*_ + *hy*_*n*_ *+ a*_*p*_ *+ α*_*l*_*x*_*l*_ *+ e*_*ijkmnpl*_ was used, where *y*_*ijkmnpl*_ was the phenotype, the overall mean, *γ*_*1*_ and *γ*_*2*_ the regression coefficients for fixed effects age (*age*_*ijkmnpl*_) and individual’s heterosis (*h*_*ijkmnpl*_), respectively, being fit as covariates, *sex*_*i*_ the fixed effect of sex (male or female), *β*_*j*_ the linear regression coefficients of the *j*^*th*^ breed, *b*_*j*_ the breed proportion of the *j*^*th*^ breed (six major breeds being Angus, Charolais, Simmental, Piedmontese, Limousin, Gelbvieh) in the *p* animal, *t*_*k*_ the fixed effect of the *k*^*th*^ trial treatment (57 groups), *g*_*m*_ the fixed effect of the *m*^*th*^ clusters (75 clusters), *hy*_*n*_ the random effect of the *n*^*th*^ herd of origin by year group (28 groups), *a*_*p*_ the random additive genetic effect of individual *p*, *x*_*l*_ the number of copies of the 2^nd^ allele (0, 1, 2) in the genotype for the *l*^*th*^ marker, *α*_*l*_ the linear regression coefficient (which is also the allele substitution effect) for the *l*^*th*^ marker, *s* the total number of SNP (38,745) included in the analysis, *e*_*ijkmnpl*_ the random residual effect. Age, heterosis, sex, trial treatment and cluster were assumed to affect all animals equally. Markers were assumed in linkage disequilibrium with quantitative trait loci (QTL) controlling the trait under investigation. Random effects *hy, a,* and *e* were assumed uncorrelated with each other. Covariance matrices of the effects were equal to $\mathrm{I\sigma}\begin{array}{c} {}_{2}\\ {}^{\mathrm{hy}} \end{array},$$A\sigma_{a}^{2},$ and $I\sigma_{e}^{2}$, respectively, where *I* was an identity matrix, and *A* the additive numerator relationship matrix among the animals. ASREML [15] was used to estimate the allele substitution effect, polygenic variance and residual variance.

Type I error rate was controlled by the false discovery rate (FDR) proposed by Benjamini and Hochberg [16]. The calculation of FDR thresholds was derived from the method proposed by Storey [17], who estimated the proportion of the *p*-values, of true null hypotheses, following a uniform distribution on the interval (0, 1). However the numbers of true null hypotheses in this study were estimated using the histogram of *p* values. The interval (0, 1) was equally partitioned into 20 bins (*e.g.* bin1 had *p* values in (0, 0.05], bin2 (0.05, 0.10]… bin20 (0.95,1]). For each trait, *n* tests were conducted with observed *p* values distributed as follows: *n*_*1*_*, n*_*2*_*… n*_*19*_*, n*_*20*_ in bin1, bin2… bin19, bin20, respectively, and $n=\sum_{t=1}^{2o}n_{i}$; *n*_*k*_ was the least number of *p* values among the bins, then 20*n*_*k*_ was the estimated number of true null hypotheses, and the FDR threshold was $\frac{\left(n-20n_{k}\right)100\%}{n}$ .

## Results and discussion

### Estimated genetic parameters

Trait means, heritabilities, genetic and phenotypic correlations, estimated from 747 animals, are presented in Tables 2, 3 and 4. Heritability estimates for HCW, BIFYLD, BF, REA, LM7D, and MRB were 0.27, 0.44, 0.35, 0.37, 0.31, and 0.62, respectively; and appeared moderate for all rib dissection traits. Back fat thickness appeared to have strong genetic correlations with other traits in Table 3 except LM7D. Yield grade had strong positive genetic correlations to fat related traits (0.65 and 0.66, BF and MRB respectively), and a slight negative correlation with REA (−0.18), because yield grade is a function of BF, MRB and REA. Genetic correlations between shear force at 7 days of post-mortem aging and other traits varied from almost null to low. This suggests that selection for back fat thickness and yield grade may not affect beef tenderness; while selection to increase carcass weight or ribeye area will potentially increase shear force at 7 days, whereas an increase in marbling score will improve beef tenderness. In our study, marbling score accounted for 8.8% of the variation in the shear force, which is close to the reports from Wulf *et al*. [18], who found that sire means for marbling accounted for 5% of the variation in sire means for tenderness; and Jones and Tatum [19], who reported a 9% of variation in tenderness was due to marbling. The estimated heritabilities for these six carcass quality traits agreed well with the estimates reported in the current literature [20].

**Table 2:** Number of observations, trait means and standard deviation

**Trait**	**Number of observations**	**Trait mean**	**SD**	**Min.**	**Max.**
BIFYLD	605	1.89	0.82	−1.50	3.90
BF (mm)	740	7.72	3.06	1.00	20.00
HCW (kg)	746	360.31	54.10	209.00	561.00
REA (sq. cm)	746	97.51	16.03	57.90	164.50
MRB	704	4.78	0.78	1.00	7.00
LM7D (kg)	679	5.12	1.41	2.20	11.30
BDFR	741	3.03	1.11	0.00	7.70
SQFR	741	9.01	2.56	0.60	16.30
INFR	741	9.17	3.46	0.00	20.50
LNR	741	55.19	12.03	15.30	81.20
BNR	741	19.48	2.28	9.80	34.10
TLFR	741	21.22	5.97	2.30	38.50

**Table 3:** Estimated heritability (diagonals), genetic (above the diagonal) and phenotypic (below the diagonal) correlations for carcass quality

	**BIFYLD**	**BF**	**HCW**	**REA**	**MRB**	**LM7D**
BIFYLD	0.44 ± 0.02	0.65 ± 0.06	0.09 ± 0.08	−0.18 ± 0.09	0.66 ± 0.06	0.04 ± 0.07
BF	0.65 ± 0.03	0.35 ± 0.04	0.42 ± 0.08	0.46 ± 0.09	0.43 ± 0.08	0.04 ± 0.07
HCW	0.16 ± 0.04	0.28 ± 0.03	0.27 ± 0.05	0.89 ± 0.02	−0.16 ± 0.10	0.21 ± 0.07
REA	−0.67 ± 0.03	−0.08 ± 0.03	0.55 ± 0.03	0.37 ± 0.03	−0.28 ± 0.10	0.15 ± 0.07
MRB	0.24 ± 0.04	0.42 ± 0.03	−0.25 ± 0.04	−0.21 ± 0.04	0.62 ± 0.09	−0.17 ± 0.09
LM7D	−0.06 ± 0.04	−0.04 ± 0.03	0.18 ± 0.04	0.15 ± 0.04	−0.25 ± 0.04	0.31 ± 0.08

**Table 4:** Estimated heritability (diagonals), genetic (above the diagonal) and phenotypic (below the diagonal) correlations for rib dissection traits

	**BDFR**	**SQFR**	**INFR**	**LNR**	**BNR**	**TLFR**
BDFR	0.30 ± 0.02	0.21 ± 0.03	0.31 ± 0.03	−0.14 ± 0.05	−0.06 ± 0.03	0.52 ± 0.02
SQFR	0.32 ± 0.03	0.48 ± 0.02	0.11 ± 0.03	−0.31 ± 0.04	−0.07 ± 0.03	0.65 ± 0.02
INFR	0.25 ± 0.04	0.18 ± 0.03	0.44 ± 0.02	−0.66 ± 0.04	−0.26 ± 0.04	0.81 ± 0.02
LNR	−0.33 ± 0.03	−0.35 ± 0.03	−0.73 ± 0.02	0.38 ± 0.04	−0.26 ± 0.04	−0.64 ± 0.04
BNR	−0.05 ± 0.03	−0.04 ± 0.03	−0.18 ± 0.03	−0.19 ± 0.03	0.33 ± 0.02	−0.23 ± 0.04
TLFR	0.55 ± 0.03	0.68 ± 0.02	0.81 ± 0.02	−0.73 ± 0.02	−0.15 ± 0.03	0.41 ± 0.02

As for rib dissection traits, heritability estimates were moderate (0.30-0.48), genetic correlations among fat related traits varied from low (0.21, BDFR and SQFR; 0.11, SQFR and INFR) to moderate (0.31, BDFR and INFR), and high (e.g. 0.52, TLFR and BDFR). Since the rib dissection traits are intrinsically related, an increase in lean component decreases fat component, and *vice versa.* Genetic correlation can suggest the level of similarity in genetic mechanisms between traits, where a higher correlation may be attributed to more positive and less negative contributions from the same loci than a lower correlation [21].

### Association analysis

**Figure 1:**
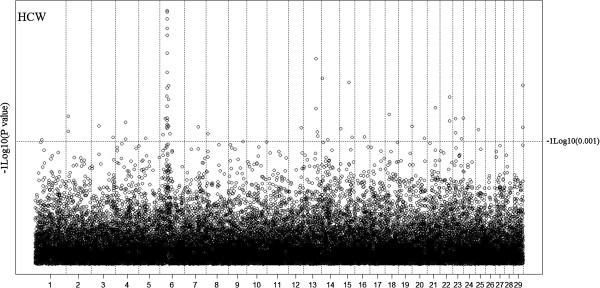
Distribution of observed P values of SNP in association with HCW.

The FDR thresholds for BF, BIF, HCW, REA, MRB, LM7D, BDFR, BNR, INFR, LNR, SQFR, TLFR were 5.12, 6.80, 6.72, 5.40, 4.92, 5.72, 7.19, 7.88, 7.19, 5.13, 5.24, and 5.18%, respectively. Number of significant SNP at different significance levels are presented in Table 5. No SNP was found significantly associated with any of the traits except for HCW at genome-wide FDR < 10%. Seven and eight SNP were in significant association with HCW at FDR < 6.72% and FDR < 10%, respectively. Seven of these SNP were positioned on chromosome 6 at 33-35 Mbp, the eighth SNP was on chromosome 13 at 61 Mbp. At a less stringent significance level of P < 0.001, 520 SNP were found significantly associated with mostly individual traits (473 SNP), and also multiple traits (47 SNP). This lends the emphasis to the point of moving from single trait SNP associations to multiple trait SNP associations. The P values of SNP in association with HCW were plotted in Figure 1. The P values of SNP in association with other traits were presented in Additional files 1, 2, 3, 4, 5, 6, 7, 8, 9, 10 and 11.

**Table 5:** Number of significant SNPs and their mean effect at various significance levels

	FDR*	**FDR < 0.1**	**P < 0.001**	**P < 0.01**	**P < 0.05**
BF	0	0	52 (2.53)	410 (1.75)	1986 (1.16)
BIF	0	0	54 (3.19)	433 (2.17)	1971 (1.44)
HCW	7 (4.90)	8 (4.80)	56 (3.06)	451 (1.85)	2084 (1.22)
REA	0	0	45 (2.63)	446 (1.78)	2060 (1.19)
MRB	0	0	34 (2.68)	391 (1.73)	1752 (1.16)
LM7D	0	0	55 (3.03)	421 (1.98)	2004 (1.30)
BDFR	0	0	52 (2.60)	392 (1.80)	1898 (1.19)
BNR	0	0	53 (2.68)	417 (1.83)	1955 (1.21)
INFR	0	0	59 (2.54)	462 (1.72)	2157 (1.14)
LNR	0	0	27 (2.63)	392 (1.69)	1864 (1.15)
SQFR	0	0	47 (2.39)	408 (1.66)	1786 (1.12)
TLFR	0	0	45 (2.51)	426 (1.67)	1988 (1.12)

* Calculated FDR threshold; Values in the parentheses are average size of SNP effect as proportion of phenotypic variance.

The allele substitution effect of significant SNP (P < 0.001), and size of the effect in terms of percentage of phenotypic variance are presented in (Additional file 12: Table S6) and (Additional file 13: Table S7), respectively. The phenotypic variance was derived as $\sigma_{p}^{2}=2\mathrm{pq}\alpha^{2}+\;\sigma_{a}^{2}+\sigma_{e}^{2}$, where $\sigma_{p}^{2}$ is the phenotypic variance, *p* the frequency of allele1, *q* the frequency of allele2, *α* the allele substitution effect, $\sigma_{a}^{2}$ the multiple additive genetic variance, $\sigma_{e}^{2}$ the residual variance. Forty-eight of these 520 SNP were located on chromosome 6, and 22 of them were in association with hot carcass weight. There were 53 SNP associated with percentage of rib bone, and 12 of them were on chromosome 20. The rest of the significant SNP were scattered over other chromosomes. In terms of effect size, these SNP accounted for 1.90 - 5.89% of the phenotypic variance, with marker ARS-BFGL-NGS-45457 on chromosome 6 explaining 5.89% of variation in HCW. Within 1.3 Mbp of this SNP were 17 others that all significantly affected HCW (P < 0.001).

Marker CAPN1_1 in the *Calpain* gene on chromosome 29 was significantly associated with shear force in this analysis, explaining 4.28% of the variation in LM7D. Similarly HAPMAP48825-BTA-60019 (BTA-60019 hereafter) on chromosome 25 accounted for 2.67% of variation in shear force but has not been documented in the literature for this trait though it is in close proximity to a QTL for beef tenderness described by Gutierrez-Gil *et al.*[22]. CAPN1_1 on BTA29 is well known for its association with tenderness, e.g. the presence of allele G at this position increased Warner Bratzler shear force in crossbred beef cattle [23,24], and in Brangus and Angus beef cattle [25]. In the current study, the presence of one copy of allele G at this location increased the Warner-Bratzler shear force by 0.50 kg. This allele was found associated with an increase in average daily gain in Brangus and Angus beef cattle [25]; and hormonal growth promotants that promote muscle growth increase the activity of the Calpastatin gene [26], resulting in less tender meat [27,28].

Kuehn *et al*. [29] estimated the frequency of allele G at CAPN1_1 in Angus (average of Angus and Red Angus), CH, GV, LM, and SM as 0.64, 0.91, 0.95, 0.92, and 0.92, respectively. The frequency of G among the 747 animals used this study was 82.48%, given that this SNP was in Hardy Weinberg equilibrium, indicating that CAPN1_1 has not been selected for in this research population. The minor allele had the highest frequency at CAPN1_1 in Angus [29], thus Angus could potentially be a good source of the minor allele to increase tenderness. Meanwhile allele T of BTA-60019, one copy of which reduced shear force by 0.32 kg in the current study, had frequencies of 0.36, 0.50, 0.10, 0.22, and 0.35 in AN (average of Angus and Red Angus), CH, GV, LM, and SM, respectively [29]. Its frequency among the 747 animals used in the current study was 30.91%. Therefore, Angus, Charolais and Simmental breeds may be used to increase the frequency of allele T at BTA-60019.

Marker CAPN1_1 on chromosome 29 was covered by three QTL for beef tenderness [30-32], and close to two QTL for tenderness [33,34]. This SNP and CAPN1_2 (at 37,544,057 bp on chromosome 29) have been described as CAPN1-316 and CAPN1-4751, respectively, by [35] in the Calpain gene. In the current study, CAPN1_2 was not significantly associated with LM7D (P > 0.05). Meanwhile marker BTA-60019 (P < 0.001) was near a QTL for tenderness reported by [30]. CAPN1_1, CAPN1_2, and BTA-60019 together explained 5% of the variation in LM7D [36]. Given LM7D is 31% heritable in the current study, these three SNP together explain approximately 16% of the additive genetic variance for this trait.

There appears to be clusters of significant SNP on chromosome 8 (37 Mbp - 40 Mbp), and 20 (4 Mbp - 5Mbp) controlling yield grade and rib eye area, as well as percentage of rib bone, respectively. In addition there was a cluster of 18 SNP on chromosome 6 (36 Mbp - 40 Mbp) significant for carcass weight and rib eye area. Twelve of them were significantly associated with birth weight and weaning weight in a separate study of the same beef cattle population [37]. Five of them (BTC-036670, BTC-034283, BTC-057761, ARS-BFGL-NGS-45457, BTC-041023) were also found to be significant for carcass weight in Japanese Black cattle [38]. This region of chromosome 6, when entered onto NCBI Map Viewer, contains 42 genes and candidate genes, involved in various functional networks, for instance nucleic acid and carbohydrate metabolism, including candidate genes *ABCG2* that affects milk production [39,40], and *SPP1* that affects yearling weight, post weaning weight gain, and HCW [41]. The chromosome 6 region was also covered by QTL for carcass weight, yearling weight and ribeye area, as well as weight gain as reported by Casas *et al*. [28], McClure *et al*. [42], and Nkrumah *et al*. [43], respectively.

Seven SNP (UA-IFASA-6538, BTC-034283, BTC-057761, ARS-BFGL-NGS-45457, BTC-041097, BTC-041023, BTC-060891) all on chromosome 6 at 37-39 Mbp, significant for HCW in this study were confirmed significant for post-weaning gain and yearling weight (P < 0.001, P < 0.001, respectively) in the USDA crossbred beef cattle population [44]. Six of these SNP (except for UA-IFASA-6538) were also in significant association with birth weight and weaning weight (both at P < 0.001), in the same USDA crossbred beef cattle data. Given high genetic correlations between carcass weight and weight gain (0.79, [45]), as well as between carcass weight and ribeye area (0.89), the 36-40 Mbp region on chromosome 6 might be a potential candidate for a search for genes influencing growth.

At less stringent significance levels, significant SNP spread out across the genome and the size of their effect shrank as the significance threshold eased up (Table 5).

### Correlation of t-test values among traits

**Figure 2:**
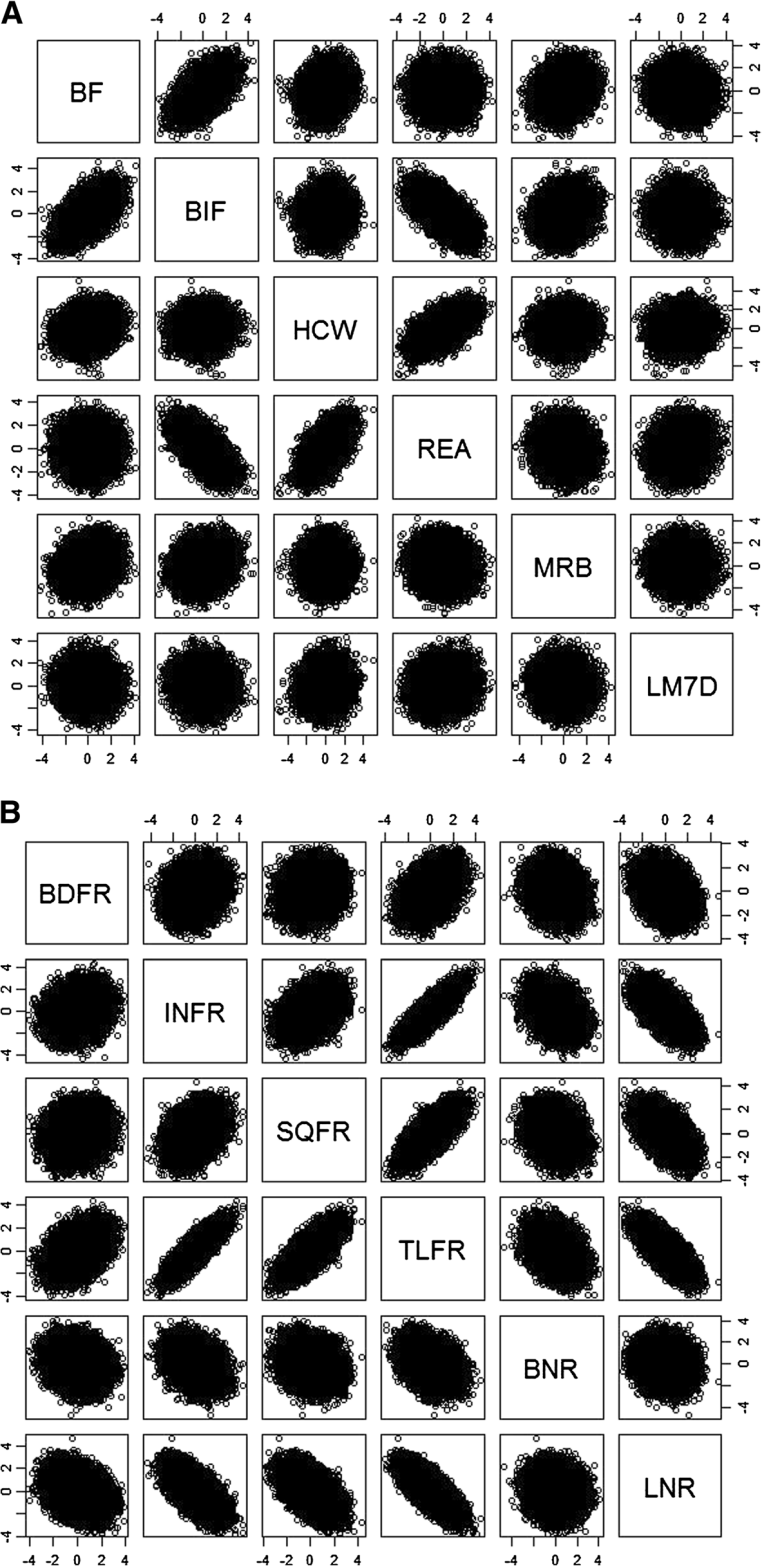
Correlation of t-test values of SNP for pairs of traits; (A) carcass quality traits; (B) rib dissection traits.

Investigating the correlations among the traits at SNP level revealed that highly correlated traits as shown in Tables 2 and 3 tend to share more SNP in same effect direction than traits with low correlation. Figure 2A and 2B show SNP correlations among the traits when all SNP are included. Strong correlations were observed between BIFYLD and BF, BIFYLD and REA, as well as HCW and REA. Most of pairs among rib dissection traits show trends (e.g. BDFR and INFR, SQFR and BNR) or strong correlations (e.g. INFR and TLFR, LNR and TLFR) in their relationships. This could be attributed to the intrinsic part-whole relationships among them. For pairs of traits that show strong, clear relationships, if genomic selection is applied to either trait in them, the corresponding trait would respond in the expected direction.

**Figure 3:**
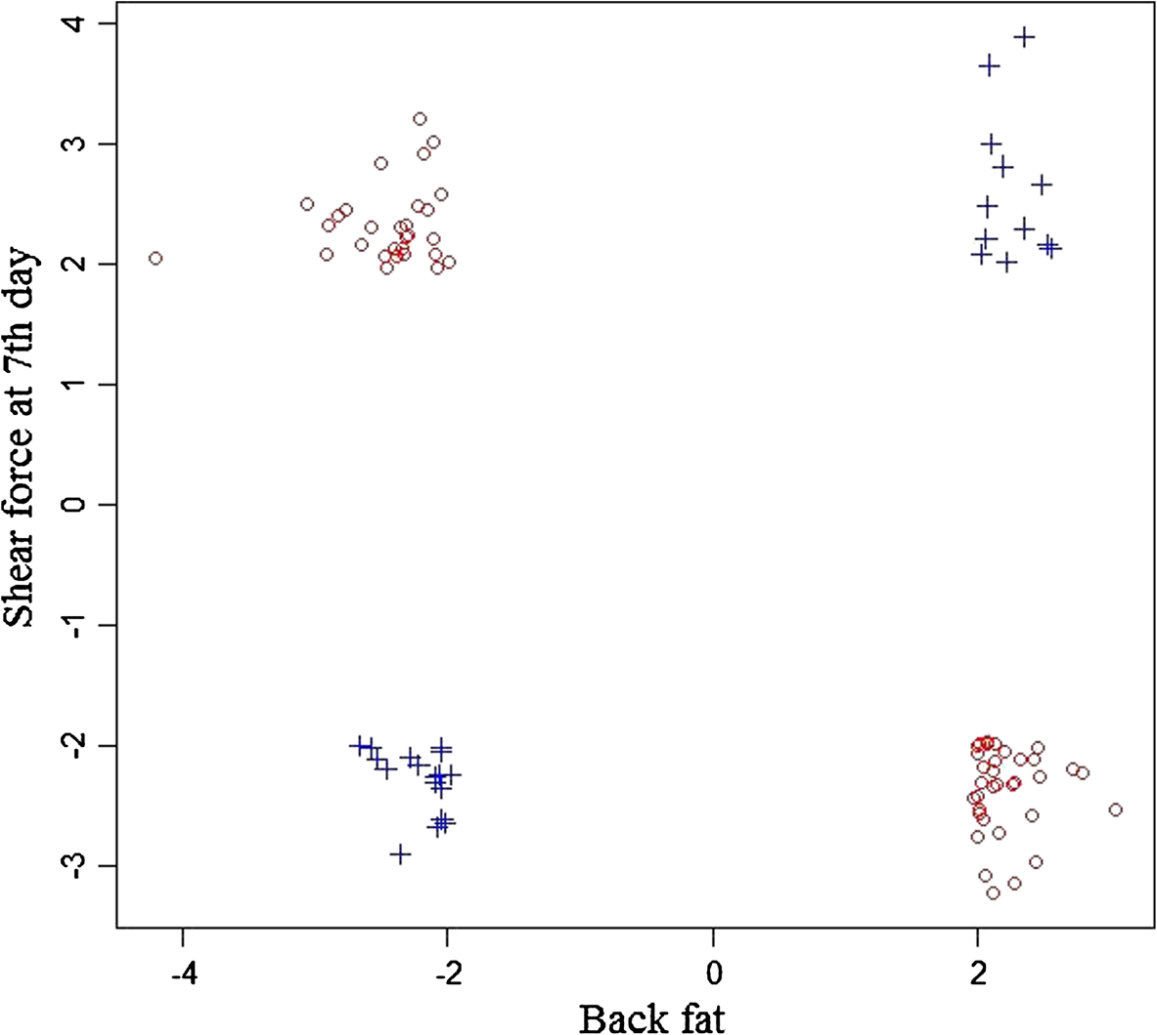
Correlation of t-test values of significant SNP (P < 0.05) for Back fat and Shear force at 7th day post-mortem.

For pairs of traits where the genetic correlations are close to zero, such as BIFYLD and LM7D, BF and LM7D, BIFYLD and HCW, their scatter plots in Figure 2A show no trend at all. However there appeared to be two groups of significant SNP (P < 0.05) involved in the relationship in those trait pairs. To demonstrate this point, Figure 3 shows 92 SNP significant for both BF and LM7D (P < 0.05). Twenty-nine of them (presented in blue crosses) affected the two traits in same direction; the rest (63 SNP presented in red circles) increased one trait while decreasing the other trait. The 63 SNP contributed negatively to the covariance between BF and LM7D. Bohren *et al*. [21] suggested that this negative contribution, together with having gene frequencies other than 0.5, is the most common factor that causes asymmetrical correlation among traits, leading to asymmetry in selection responses. Where this applies, selection of subsets of SNP for any applications should be carried out with cautions because chosen SNP may affect multiple traits.

## Conclusions

This study took a genome-wide association approach to seek regions across the bovine genome that could potentially lead to the identification of causative mutations underlying variation in carcass quality traits in beef cattle. Apart from SNP found significantly associated with the twelve traits reported in this study, chromosome 6 appeared to contain regions for future investigations into quantitative trait nucleotides that control growth and weight in beef cattle. Marker BTA-60019 on chromosome 25 needs further research into its contribution to the variation in beef tenderness.

Single nucleotide polymorphisms that were significantly associated with the traits may hold real association with genes controlling those traits. Genomic regions containing such SNP, if densely genotyped or sequenced, could be used to identify causal mutations underlying trait variation, and thus help facilitate the selection process to improve the trait of interest. An example for this is the validation of the effect of the CC at *CAPN1* gene resulting more tender meat than homozygous TT in a diverse range of breeds.

Traits analyzed in the current study had heritability estimates from low to high, thus genetic improvement of the trait using traditional selection would be possible where phenotypes are readily available as part of mainstream recording. For traits that show very low genetic correlations, the current study revealed that such traits may be mainly controlled by two groups of genes, one increasing both traits, the other increasing one trait while decreasing the other trait at the same time. Where this applies, a carefully chosen subset of SNP during selection may lead to improvement in both traits.

## Abbreviations

QTN: Quantitative trait nucleotide, referring to a causative mutation that accounts for differences in phenotype; QTL: Quantitative trait loci, referring to regions of chromosome that contains or links to genes underlying a quantitative trait; SNP: Single nucleotide polymorphism; IMPS: Institutional meat purchase specifications; HCW: Hot carcass weight; BF: Back fat thickness; REA: Ribeye area; MRB: Marbling score; BIFYLD: Lean yield grade by Beef Improvement Federation; LM7D: Warner-Bratzler shear force 7-day post mortem; BDFR: Body cavity fat rate; LNR: Lean rate; BNR: Bone rate; INFR: Intermuscular fat rate; SQFR: Subcuraneous fat rate; TLFR: Total fat rate; FDR: False discovery rate; BTA: *Bos taurus* autosome; EPD: Expected progeny difference; AN: Angus; CH: Charolais; SM: Simmental; PI: Piedmontese; LM: Limousin; GV: Gelbvieh; EBRC: Elora Beef Research Centre; NLARS: New Liskeard Agriculture Research Station; KAP: Kapuskasing experimental farm; MAF: Minor allele frequency

## Authors’ contributions

DL participated in designing the study, carried out the analysis and interpretation of data, initiated and revised the manuscript; SPM sought funding for the project, participated in designing the study, and drafting the manuscript; MS designed the software and participated in drafting the manuscript; MK participated in designing the study and drafting the manuscript; GVV participated in data management and drafting the manuscript; IM helped with trait measurement; ZW, SM, GP sought funding for the project, and helped draft the manuscript. All authors read and approved the final manuscript.

## Supplementary Material

Additional file 1 Distribution of observed P values of SNP in association with Back fat. Click here for file

Additional file 2 Distribution of observed P values of SNP in association with BIF yield grade. Click here for file

Additional file 3 Distribution of observed P values of SNP in association with Ribeye area. Click here for file

Additional file 4 Distribution of observed P values of SNP in association with Marbling. Click here for file

Additional file 5 Distribution of observed P values of SNP in association with Shear force at 7th day post-mortem. Click here for file

Additional file 6 Distribution of observed P values of SNP in association with Body cavity fat percentage of rib. Click here for file

Additional file 7 Distribution of observed P values of SNP in association with Lean percentage of rib. Click here for file

Additional file 8 Distribution of observed P values of SNP in association with Bone percentage of rib. Click here for file

Additional file 9 Distribution of observed P values of SNP in association with Intermuscular fat percentage of rib. Click here for file

Additional file 10 Distribution of observed P values of SNP in association with Subcutaneous fat percentage of rib. Click here for file

Additional file 11 Distribution of observed P values of SNP in association with Total fat percentage of rib. Click here for file

Additional file 12: Table S6 Allele substitution effect of significant SNP (at P < 0.001). Click here for file

Additional file 13: Table S7 Size of SNP effect expressed as percentage of phenotypic variance. Click here for file
