# Supplementary figures and images for: Genome-wide association analyses for carcass quality in crossbred beef cattle
Source: BMC Genet. 2013 Sep 11;14:80. doi: 10.1186/1471-2156-14-80 (PMC3827924; doi:10.1186/1471-2156-14-80)

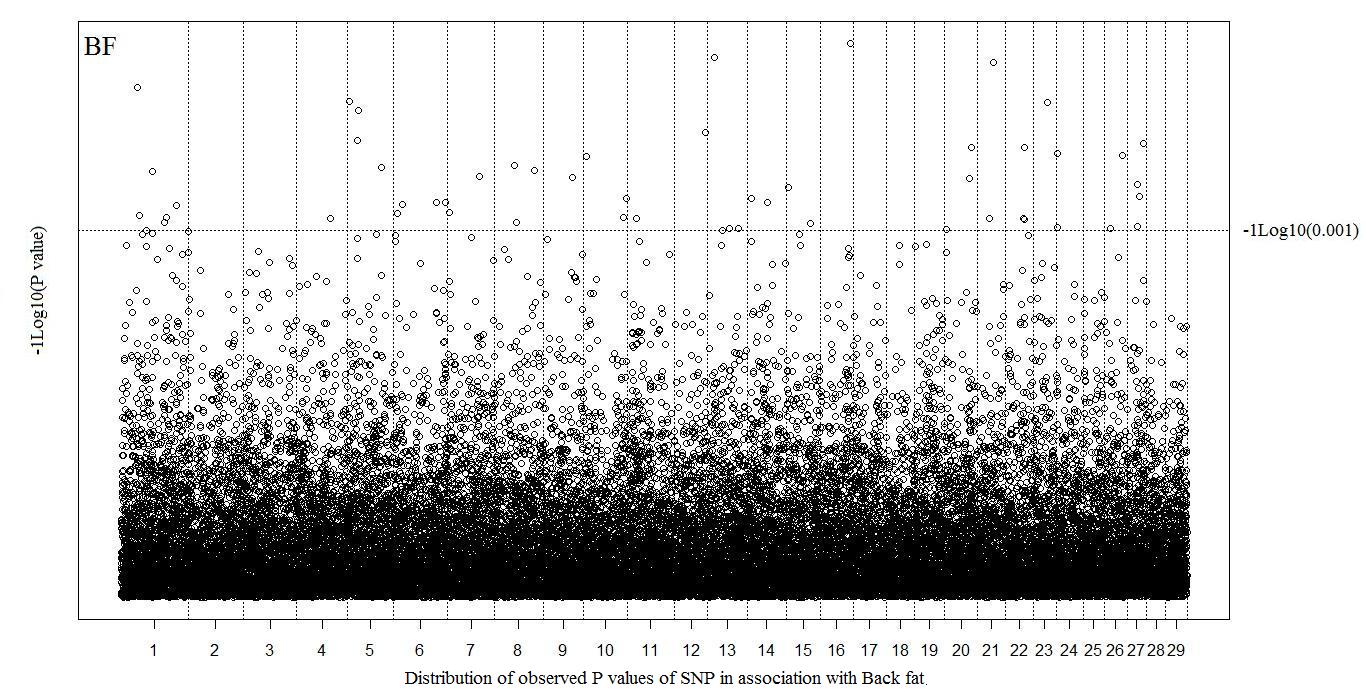

Supplement: Additional file 1 — Distribution of observed P values of SNP in association with Back fat. [file 1471-2156-14-80-S1.jpeg]

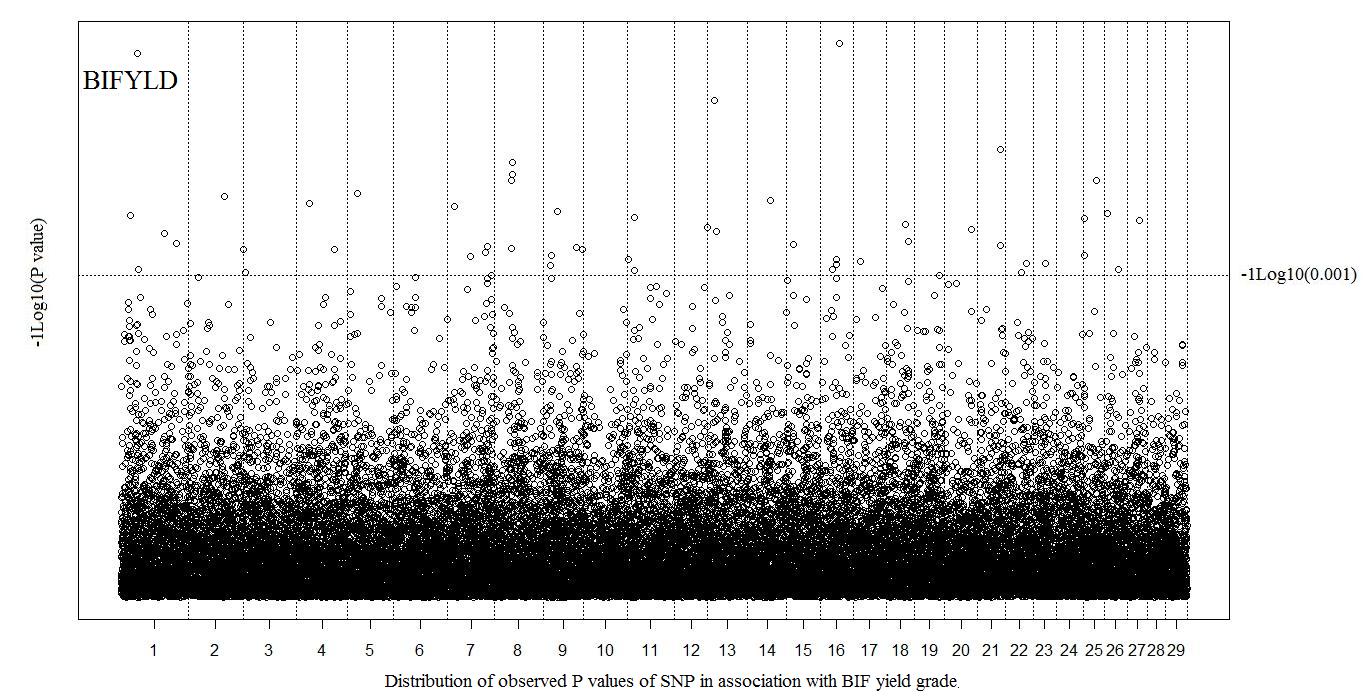

Supplement: Additional file 2 — Distribution of observed P values of SNP in association with BIF yield grade. [file 1471-2156-14-80-S2.jpeg]

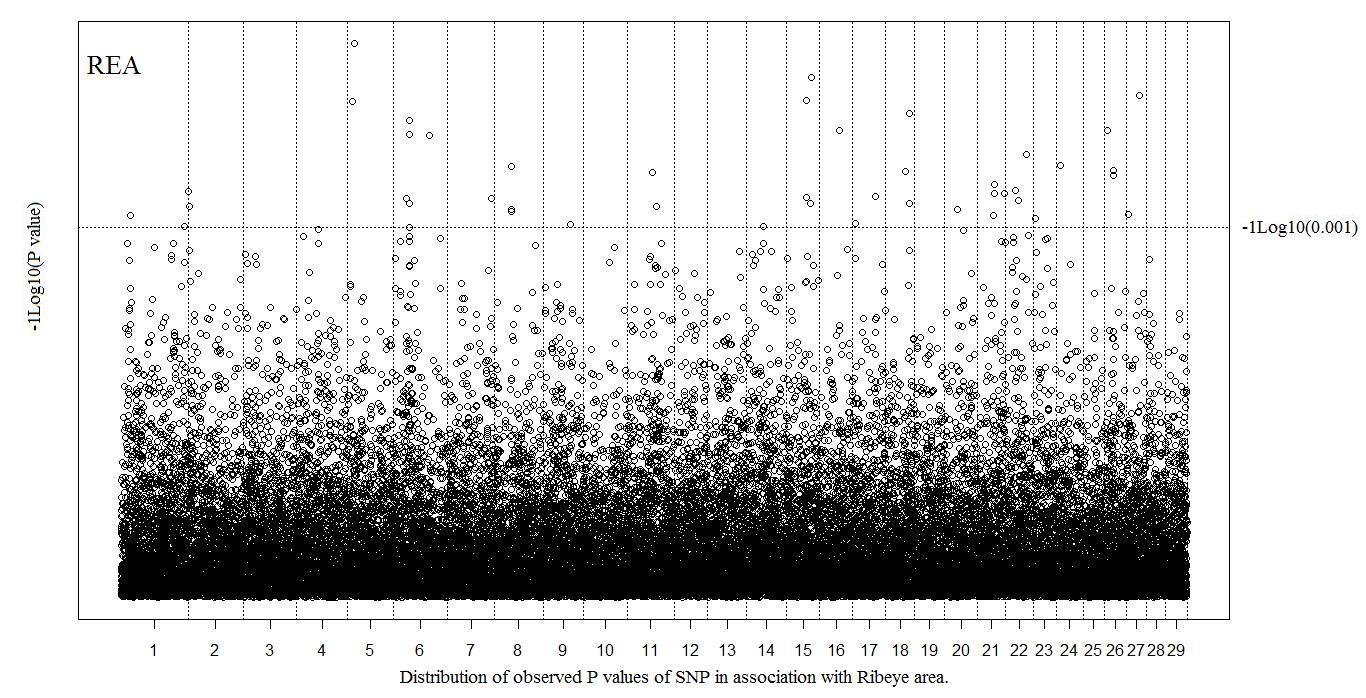

Supplement: Additional file 3 — Distribution of observed P values of SNP in association with Ribeye area. [file 1471-2156-14-80-S3.jpeg]

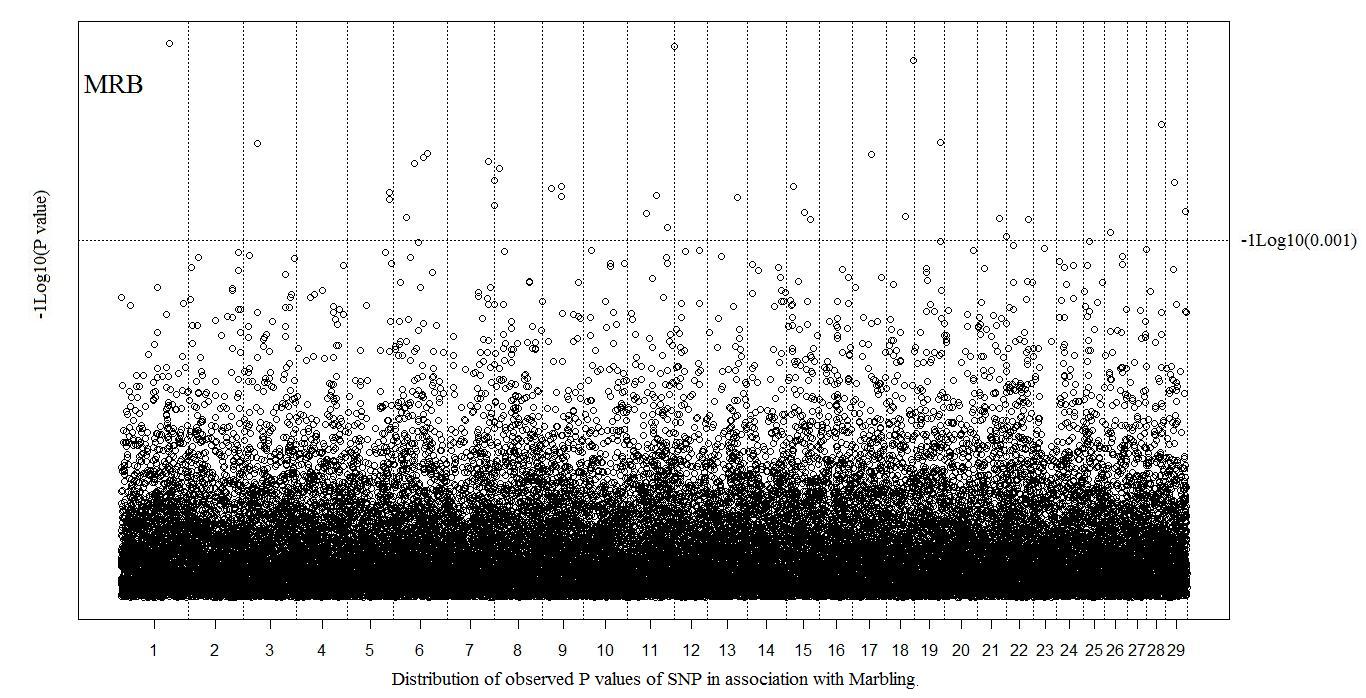

Supplement: Additional file 4 — Distribution of observed P values of SNP in association with Marbling. [file 1471-2156-14-80-S4.jpeg]

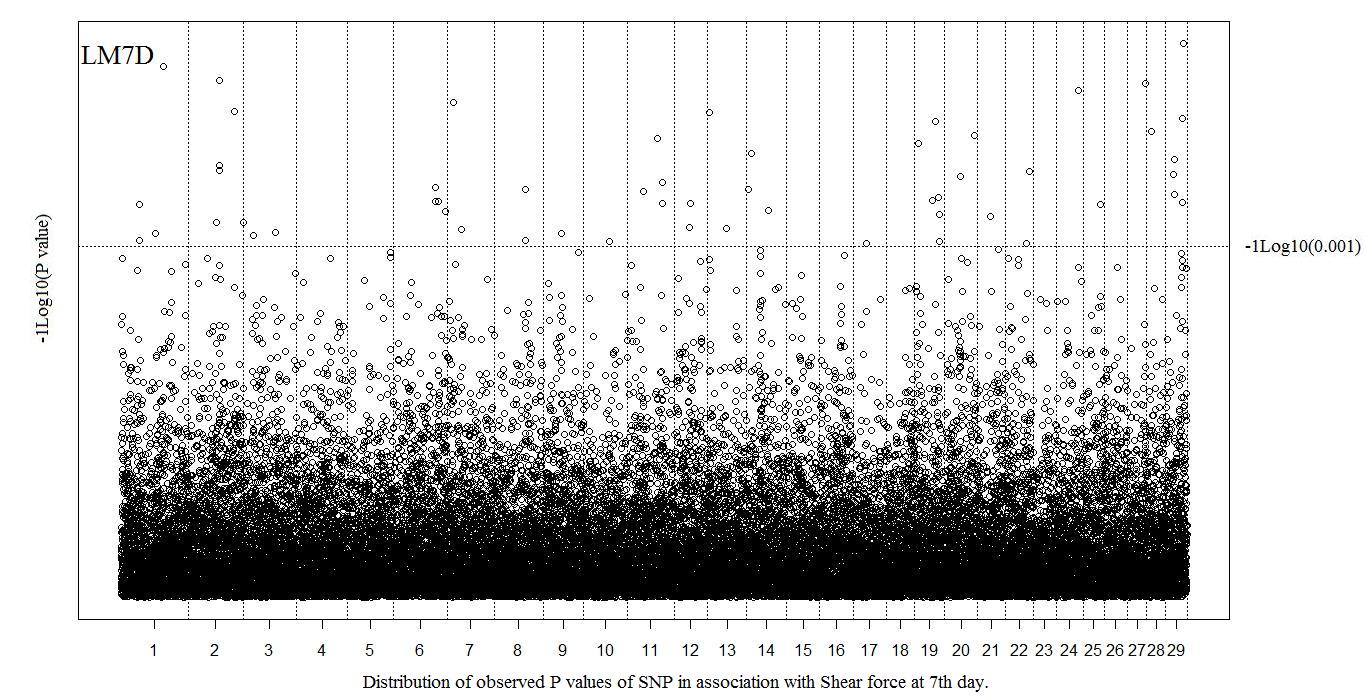

Supplement: Additional file 5 — Distribution of observed P values of SNP in association with Shear force at 7th day post-mortem. [file 1471-2156-14-80-S5.jpeg]

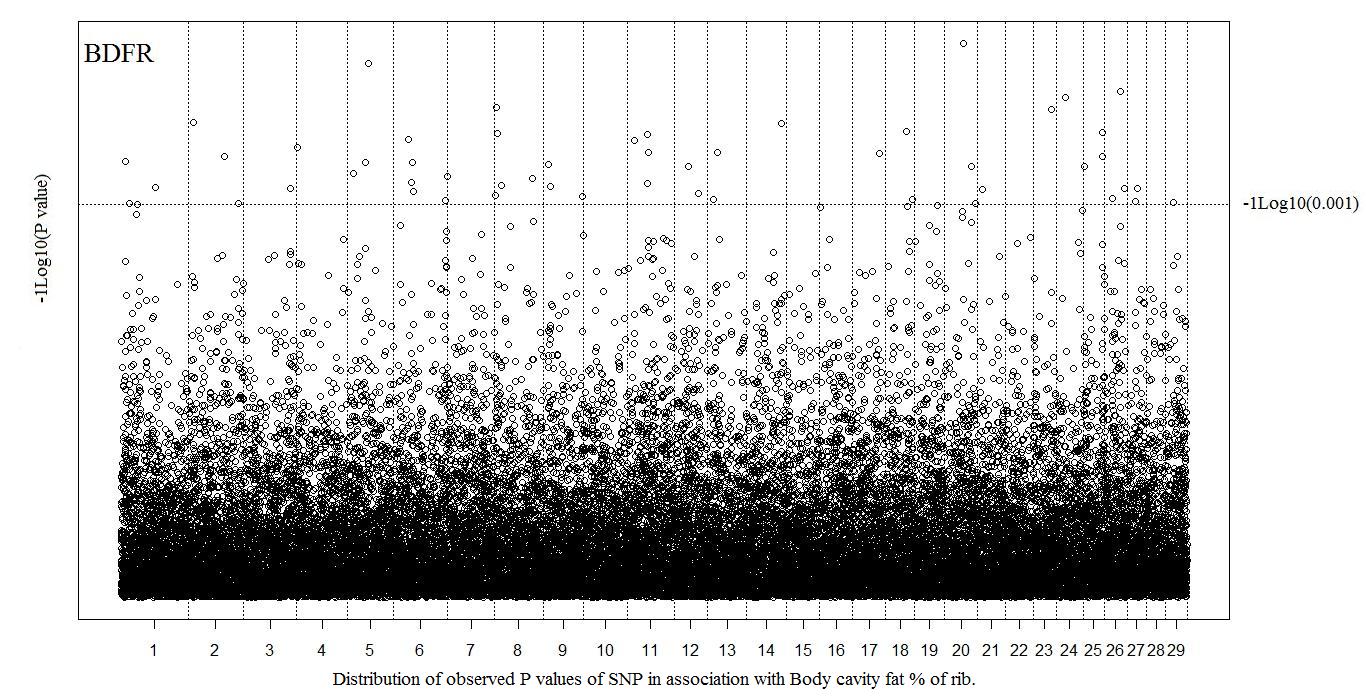

Supplement: Additional file 6 — Distribution of observed P values of SNP in association with Body cavity fat percentage of rib. [file 1471-2156-14-80-S6.jpeg]

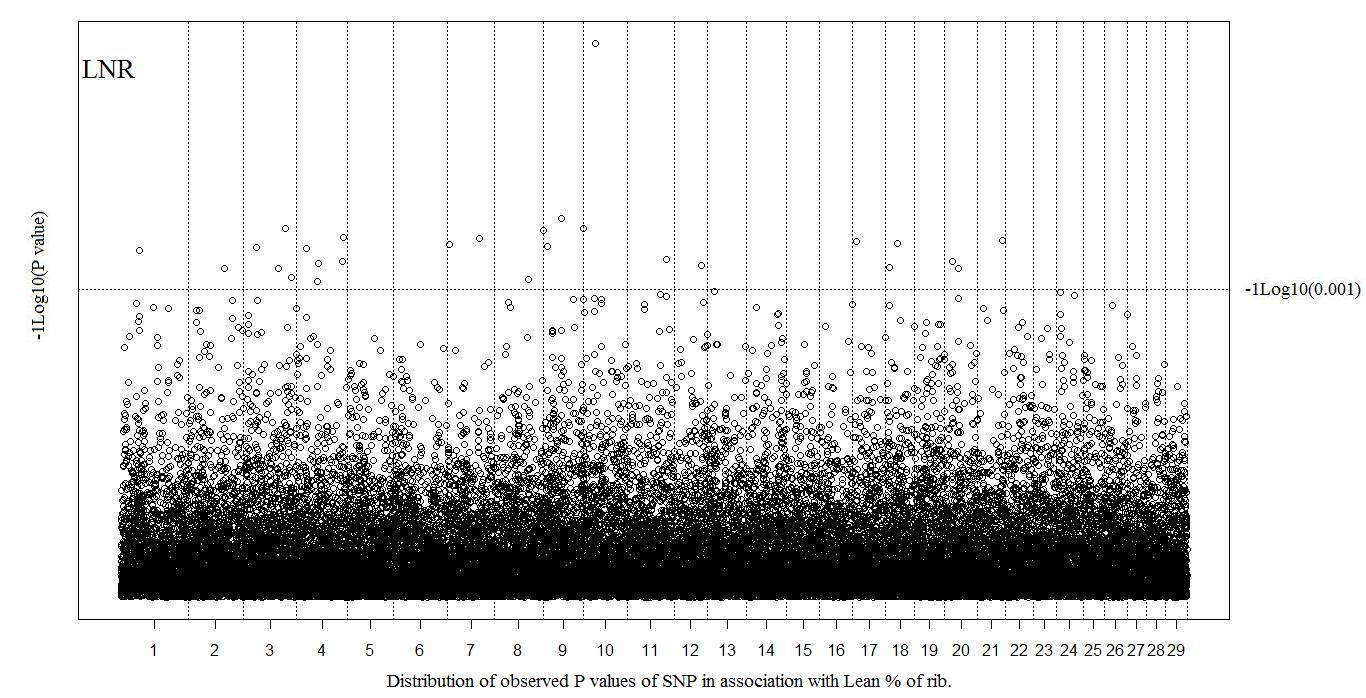

Supplement: Additional file 7 — Distribution of observed P values of SNP in association with Lean percentage of rib. [file 1471-2156-14-80-S7.jpeg]

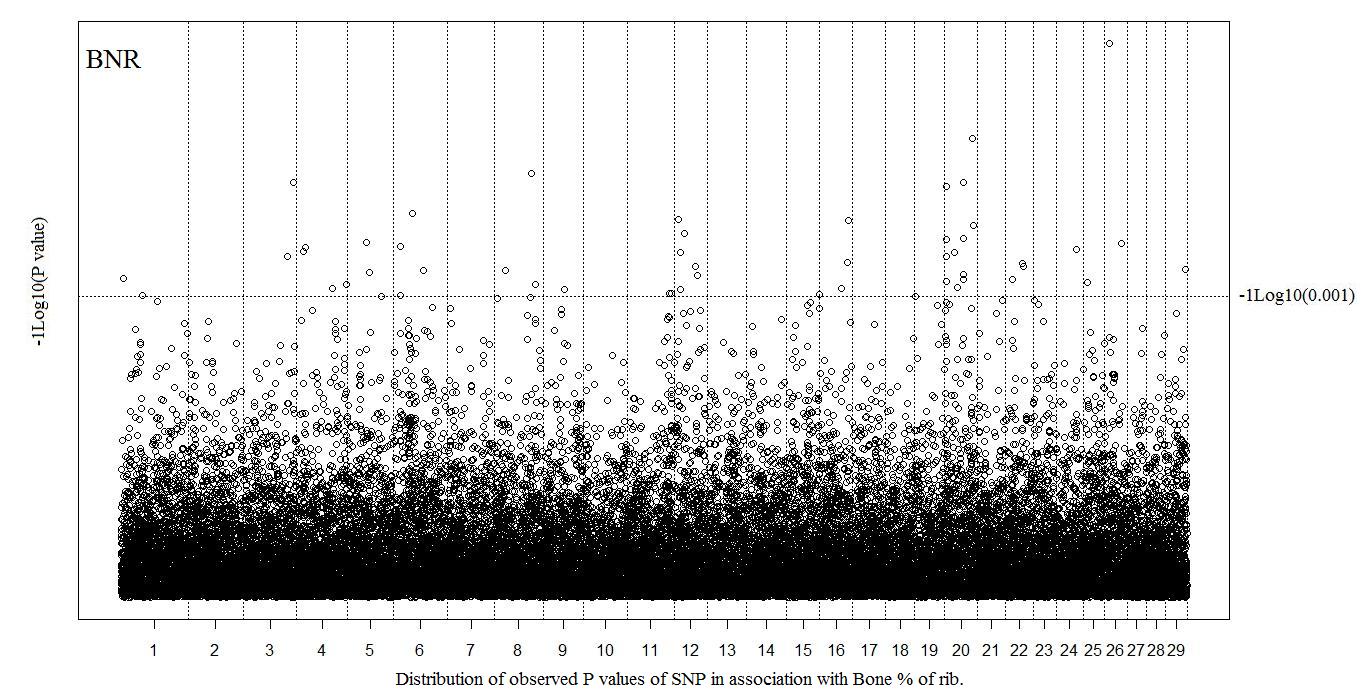

Supplement: Additional file 8 — Distribution of observed P values of SNP in association with Bone percentage of rib. [file 1471-2156-14-80-S8.jpeg]

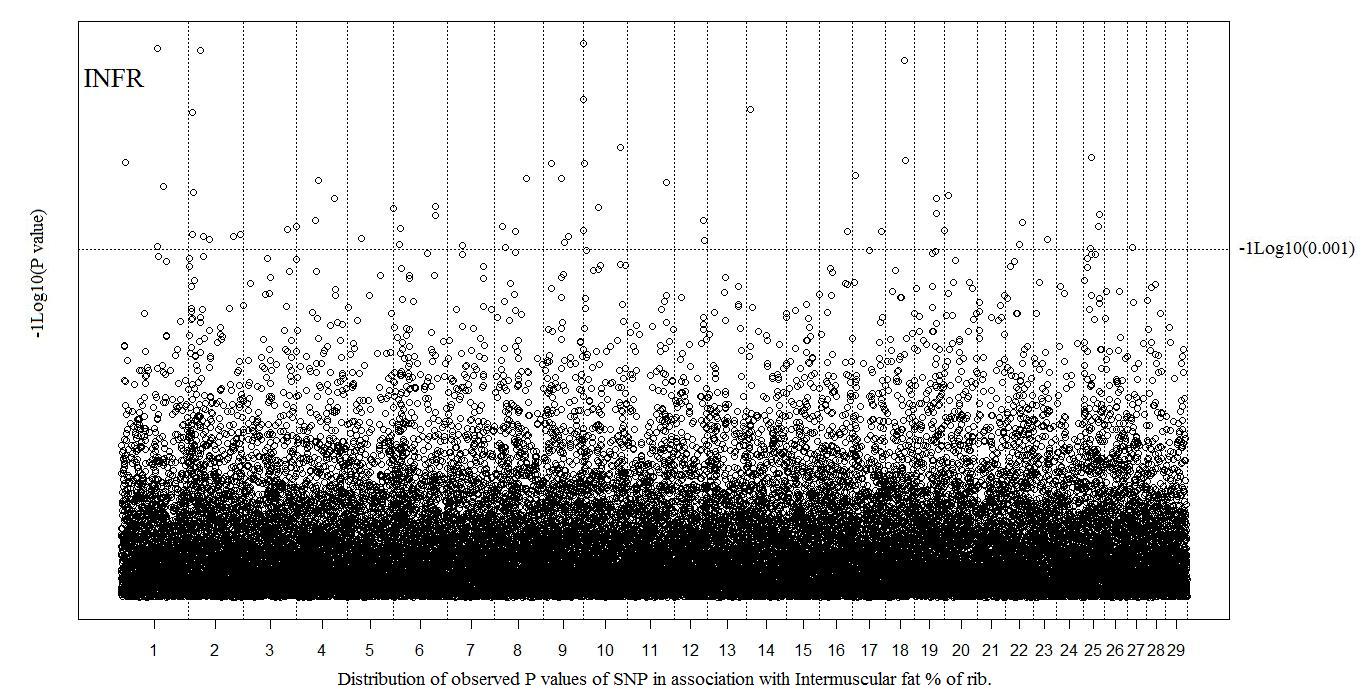

Supplement: Additional file 9 — Distribution of observed P values of SNP in association with Intermuscular fat percentage of rib. [file 1471-2156-14-80-S9.jpeg]

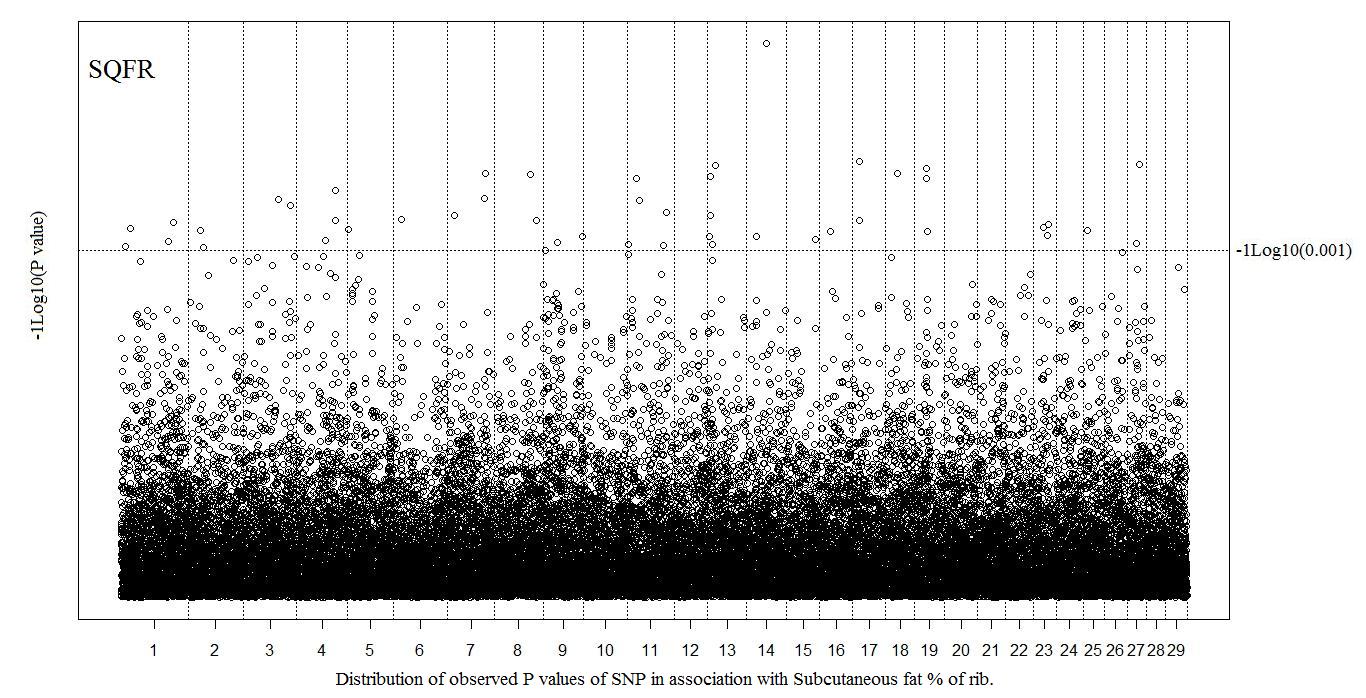

Supplement: Additional file 10 — Distribution of observed P values of SNP in association with Subcutaneous fat percentage of rib. [file 1471-2156-14-80-S10.jpeg]

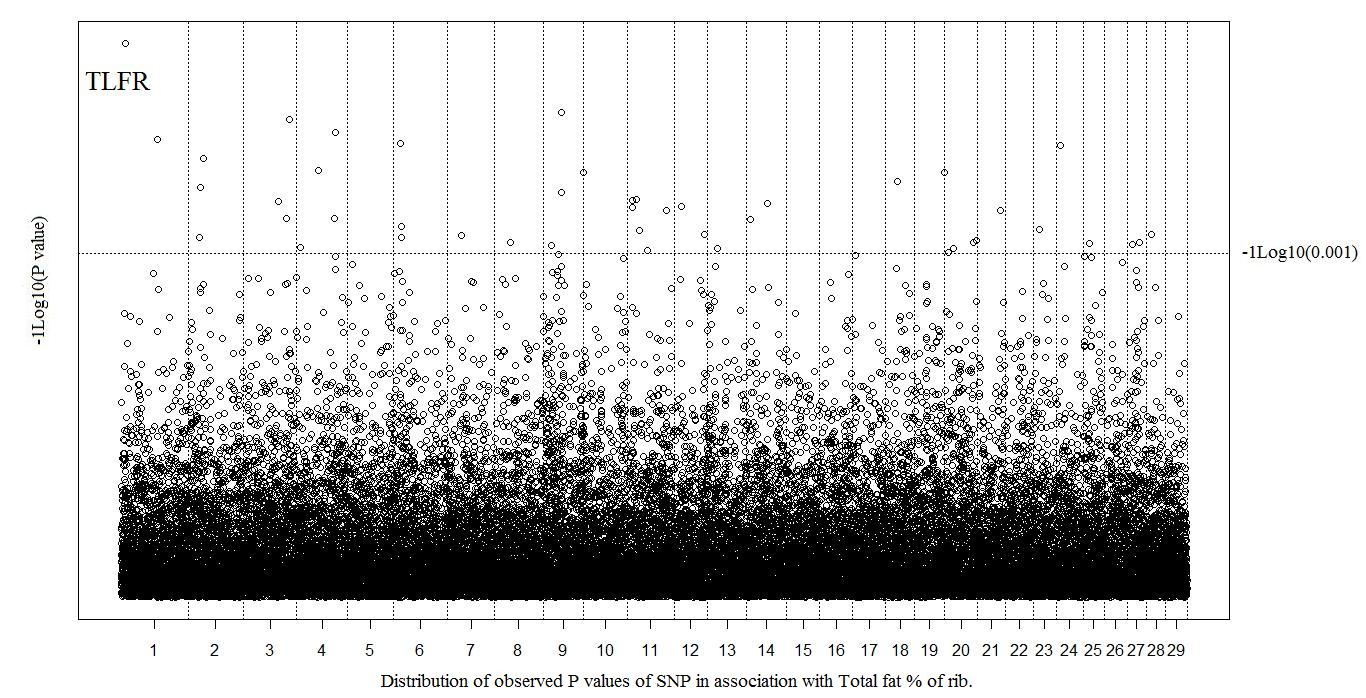

Supplement: Additional file 11 — Distribution of observed P values of SNP in association with Total fat percentage of rib. [file 1471-2156-14-80-S11.jpeg]
