# Supplementary material for: Genome-wide association analyses for carcass quality in crossbred beef cattle
Source: BMC Genet. 2013 Sep 11;14:80. doi: 10.1186/1471-2156-14-80 (PMC3827924; doi:10.1186/1471-2156-14-80)
Supplement: Additional file 12: Table S6 — Allele substitution effect of significant SNP (at P < 0.001). [file 1471-2156-14-80-S12.docx]

| Table S6. Allele substitution effect of significant SNP (at P < 0.001) | | | | | | | | | | | | | | |
| --- | --- | --- | --- | --- | --- | --- | --- | --- | --- | --- | --- | --- | --- | --- |
| SNP name | Bta | Position (bp) | BF | BIF | HCW | REA | MRB | LM7D | BDFR | BNR | INFR | LNR | SQFR | TLFR |
| HAPMAP58931-RS29010773 | 1 | 5741816 |  |  |  |  |  |  |  | 2.41 |  |  |  |  |
| HAPMAP54753-RS29013987 | 1 | 8240375 |  |  |  |  |  |  |  |  | 1.65 |  | 0.76 | 6.07 |
| BTA-38496-NO-RS | 1 | 9404794 |  |  |  |  |  |  | 0.19 |  |  |  |  |  |
| BTB-01511655 | 1 | 18052292 |  |  |  |  |  |  |  |  |  |  | -0.89 |  |
| BTB-01116164 | 1 | 21656798 |  | -0.22 |  |  |  |  |  |  |  |  |  |  |
| ARS-BFGL-NGS-51361 | 1 | 21864313 |  |  |  | 4.55 |  |  |  |  |  |  |  |  |
| BTB-00010474 | 1 | 24757983 |  |  |  |  |  |  | -0.19 |  |  |  |  |  |
| BTA-16749-NO-RS | 1 | 38352766 |  |  | 8.69 |  |  |  |  |  |  |  |  |  |
| ARS-BFGL-NGS-63816 | 1 | 41756440 |  | 0.18 |  |  |  |  |  |  |  |  |  |  |
| BTA-27636-NO-RS | 1 | 42434198 | 0.93 |  |  |  |  |  |  |  |  |  |  |  |
| BTA-89708-NO-RS | 1 | 43293171 |  | 0.14 |  |  |  |  |  |  |  |  |  |  |
| HAPMAP57446-RS29023998 | 1 | 49393074 |  |  |  |  |  | 0.47 |  |  |  |  |  |  |
| BTA-114651-NO-RS | 1 | 50252331 | 0.50 |  |  |  |  |  |  |  |  |  |  |  |
| HAPMAP41715-BTA-100026 | 1 | 50320575 |  |  |  |  |  | -0.31 |  |  |  |  |  |  |
| BTB-01165120 | 1 | 50465233 |  |  |  |  |  |  |  |  |  | 3.86 |  |  |
| ARS-BFGL-NGS-107096 | 1 | 53753590 |  |  |  |  |  |  |  | -2.13 |  |  |  |  |
| HAPMAP26500-BTA-37512 | 1 | 78763254 | 0.63 |  |  |  |  |  |  |  |  |  |  |  |
| BTA-38441-NO-RS | 1 | 80875014 |  |  |  |  |  | 0.30 |  |  |  |  |  |  |
| ARS-BFGL-NGS-26946 | 1 | 80974985 |  |  |  |  |  |  | 0.18 |  |  |  |  |  |
| BTB-00036784 | 1 | 86888776 |  |  |  |  |  |  |  |  | 1.83 |  |  | 5.29 |
| HAPMAP45277-BTA-38474 | 1 | 86910584 |  |  |  |  |  |  |  |  | -1.42 |  |  |  |
| ARS-BFGL-NGS-29866 | 1 | 98196332 |  |  |  |  |  | 0.36 |  |  |  |  |  |  |
| HAPMAP43856-BTA-44227 | 1 | 99193415 |  |  |  |  |  |  |  |  | 1.65 |  |  |  |
| BTB-01631727 | 1 | 100800828 | -0.60 | -0.17 |  |  |  |  |  |  |  |  |  |  |
| BTA-39419-NO-RS | 1 | 103618079 | 0.51 |  |  |  |  |  |  |  |  |  |  |  |
| ARS-BFGL-NGS-37145 | 1 | 115072563 |  |  |  |  |  |  |  |  |  |  | 0.89 |  |
| HAPMAP42953-BTA-48231 | 1 | 118532290 |  |  |  |  | -0.18 |  |  |  |  |  |  |  |
| HAPMAP31516-BTA-161480 | 1 | 124416832 |  |  |  |  |  |  |  |  |  |  | -1.02 |  |
| ARS-BFGL-NGS-1834 | 1 | 132696696 |  | -0.15 |  |  |  |  |  |  |  |  |  |  |
| SNP name | Bta | Position (bp) | BF | BIF | HCW | REA | MRB | LM7D | BDFR | BNR | INFR | LNR | SQFR | TLFR |
| ARS-BFGL-BAC-11057 | 1 | 134030804 | -0.54 |  |  |  |  |  |  |  |  |  |  |  |
| HAPMAP53066-RS29026782 | 1 | 152345820 |  |  |  | 4.73 |  |  |  |  |  |  |  |  |
| BTA-109914-NO-RS | 2 | 547782 |  |  |  | 5.00 |  |  |  |  |  |  |  |  |
| ARS-BFGL-BAC-2619 | 2 | 5780265 |  |  |  | 4.99 |  |  |  |  |  |  |  |  |
| BFGL-NGS-114790 | 2 | 9785462 |  |  |  |  |  |  |  |  | 1.57 |  |  |  |
| HAPMAP46046-BTA-114001 | 2 | 12277461 |  |  |  |  |  |  |  |  | 1.45 |  |  |  |
| ARS-BFGL-NGS-74668 | 2 | 13941524 |  |  |  |  |  |  | 0.23 |  |  |  |  |  |
| BFGL-NGS-112128 | 2 | 14336331 |  |  | -10.28 |  |  |  |  |  |  |  |  |  |
| HAPMAP47793-BTA-97444 | 2 | 14360005 |  |  | -8.45 |  |  |  |  |  |  |  |  |  |
| BTA-109896-NO-RS | 2 | 16238330 |  |  |  |  |  |  |  |  | 2.01 |  |  |  |
| BTA-47074-NO-RS | 2 | 27863911 |  |  |  |  |  |  |  |  |  |  |  | 5.53 |
| HAPMAP44243-BTA-47059 | 2 | 28161110 |  |  |  |  |  |  |  |  | -2.31 |  |  | -6.29 |
| HAPMAP57743-RS29026473 | 2 | 28376835 |  |  |  |  |  |  |  |  |  |  | 0.77 |  |
| BTA-96624-NO-RS | 2 | 28758897 |  |  |  |  |  |  |  |  |  |  | -0.71 | -4.98 |
| BTB-01494156 | 2 | 28923176 |  |  |  |  |  |  |  |  | 1.65 |  |  |  |
| ARS-BFGL-NGS-98960 | 2 | 51385485 |  |  |  |  |  |  |  |  | -2.24 |  |  |  |
| HAPMAP60758-RS29010833 | 2 | 66885868 |  |  |  |  |  | 0.30 |  |  |  |  |  |  |
| ARS-BFGL-NGS-38374 | 2 | 74388391 |  |  |  |  |  | -0.48 |  |  |  |  |  |  |
| HAPMAP23997-BTA-145416 | 2 | 74422326 |  |  |  |  |  | 0.36 |  |  |  |  |  |  |
| HAPMAP32014-BTA-108899 | 2 | 74743086 |  |  |  |  |  | 0.31 |  |  |  |  |  |  |
| HAPMAP39700-BTA-93776 | 2 | 88925365 |  | -0.15 |  |  |  |  |  |  |  | 2.94 |  |  |
| HAPMAP41888-BTA-49091 | 2 | 116433943 |  |  |  |  |  | -0.37 |  |  |  |  |  |  |
| BTA-95472-NO-RS | 2 | 117233854 |  |  |  |  |  |  |  |  | 1.81 |  |  |  |
| ARS-BFGL-NGS-40911 | 2 | 129554709 |  |  |  |  |  |  | 0.24 |  |  |  |  |  |
| ARS-BFGL-NGS-22876 | 2 | 133403110 |  |  |  |  |  |  |  |  | -1.43 |  |  |  |
| ARS-BFGL-NGS-15882 | 3 | 991777 |  | 0.13 |  |  |  |  |  |  |  |  |  |  |
| ARS-BFGL-NGS-104680 | 3 | 1079354 |  |  |  |  |  | 0.32 |  |  |  |  |  |  |
| ARS-BFGL-NGS-70744 | 3 | 10422377 |  | 0.15 |  |  |  |  |  |  |  |  |  |  |
| ARS-BFGL-NGS-105333 | 3 | 27170513 |  |  |  |  |  | -0.34 |  |  |  |  |  |  |
| ARS-BFGL-NGS-83049 | 3 | 34817679 |  |  |  |  |  |  |  |  |  | -3.69 |  |  |
| SNP name | Bta | Position (bp) | BF | BIF | HCW | REA | MRB | LM7D | BDFR | BNR | INFR | LNR | SQFR | TLFR |
| BTB-01405537 | 3 | 40364628 |  |  |  |  | -0.22 |  |  |  |  |  |  |  |
| BTB-02008212 | 3 | 46771130 |  |  | 13.03 |  |  |  |  |  |  |  |  |  |
| UA-IFASA-2622 | 3 | 79069350 |  |  |  |  |  | -0.30 |  |  |  |  |  |  |
| BTB-01832443 | 3 | 81379707 |  |  |  |  |  |  |  |  |  | 3.00 | -0.81 | -4.96 |
| HAPMAP48025-BTA-62581 | 3 | 99387696 |  |  |  |  |  |  |  |  |  | 3.59 |  |  |
| BFGL-NGS-116359 | 3 | 100184301 |  |  |  |  |  |  |  |  |  |  |  | -5.32 |
| BFGL-NGS-109696 | 3 | 102092165 |  |  |  |  |  |  |  |  | -1.48 |  |  |  |
| ARS-BFGL-NGS-30263 | 3 | 103650423 |  |  |  |  |  |  |  |  |  |  |  | -8.19 |
| BTB-00154000 | 3 | 105064402 |  |  |  |  |  |  |  | -3.11 |  |  |  |  |
| ARS-BFGL-NGS-30729 | 3 | 108311829 |  |  |  |  |  |  |  |  |  |  | -0.78 |  |
| BFGL-NGS-114817 | 3 | 109619527 |  |  |  |  |  |  | -0.18 |  |  |  |  |  |
| ARS-BFGL-NGS-15623 | 3 | 111276607 |  |  |  |  |  |  |  |  |  | 2.98 |  |  |
| ARS-BFGL-NGS-11532 | 3 | 111669871 |  |  | 8.61 |  |  |  |  |  |  |  |  |  |
| HAPMAP55088-RS29012399 | 3 | 114730114 |  |  |  |  |  |  |  | -2.47 |  |  |  |  |
| ARS-BFGL-NGS-6917 | 3 | 121087285 |  |  |  |  |  |  |  |  | -1.97 |  |  |  |
| HAPMAP51156-BTA-116504 | 4 | 8898535 |  |  |  |  |  |  |  |  |  |  |  | 5.18 |
| BFGL-NGS-119147 | 4 | 15279059 |  |  |  |  |  |  |  | -2.07 |  |  |  |  |
| HAPMAP38534-BTA-85405 | 4 | 17556319 |  |  |  |  |  |  |  |  |  | -3.17 |  |  |
| ARS-BFGL-NGS-4793 | 4 | 19643696 |  |  |  |  |  |  | 0.25 |  |  |  |  |  |
| BTB-00170600 | 4 | 27101116 |  | 0.19 |  |  |  |  |  |  |  |  |  |  |
| ARS-BFGL-NGS-3245 | 4 | 38435198 |  |  |  |  |  |  |  | 2.03 |  |  |  |  |
| ARS-BFGL-NGS-90711 | 4 | 45577225 |  |  |  |  |  |  |  |  | 1.50 |  |  |  |
| BFGL-NGS-114296 | 4 | 53143855 |  |  | -9.05 |  |  |  |  |  |  |  |  |  |
| BTB-01065966 | 4 | 53240815 |  |  | 8.18 |  |  |  |  |  |  |  |  |  |
| BTB-01066381 | 4 | 53777012 |  |  |  |  |  |  |  |  | 1.82 | -3.45 |  | 5.92 |
| BTA-110705-NO-RS | 4 | 55313701 |  |  |  |  |  |  |  |  |  | 2.87 |  |  |
| HAPMAP36554-NPY_499F3-SNP1 | 4 | 72071212 |  |  |  |  |  |  |  |  |  |  | 0.84 |  |
| BTB-01928726 | 4 | 80280325 | -0.50 |  |  |  |  |  |  |  |  |  |  |  |
| BTB-01032336 | 4 | 86540790 |  |  |  |  |  |  |  | 1.94 |  |  |  |  |
| HAPMAP50910-BTA-18270 | 4 | 90275283 |  |  |  |  |  |  |  |  |  |  | -0.84 |  |
| SNP name | Bta | Position (bp) | BF | BIF | HCW | REA | MRB | LM7D | BDFR | BNR | INFR | LNR | SQFR | TLFR |
| ARS-BFGL-NGS-95211 | 4 | 90314494 |  |  |  |  |  |  |  |  |  |  | -0.81 | -5.39 |
| BTB-02000619 | 4 | 92027885 |  |  |  |  |  |  |  |  | 1.75 |  |  | 5.46 |
| BFGL-NGS-118100 | 4 | 92141993 |  | -0.13 |  |  |  |  |  |  |  |  |  |  |
| BTB-00208231 | 4 | 110806820 |  |  |  |  |  |  |  |  |  | 2.89 |  |  |
| ARS-BFGL-NGS-61198 | 4 | 112474006 |  |  |  |  |  |  |  |  |  | -3.06 |  |  |
| BTB-01493530 | 4 | 116292574 |  |  |  |  |  |  |  | -1.96 |  |  |  |  |
| BTB-01735758 | 5 | 1087211 |  |  |  |  |  |  |  |  |  |  | 0.74 |  |
| HAPMAP31056-BTA-114319 | 5 | 1964556 | -0.63 |  |  |  |  |  |  |  |  |  |  |  |
| BTB-00220941 | 5 | 8435987 |  |  |  | 5.74 |  |  |  |  |  |  |  |  |
| HAPMAP52005-BTA-75510 | 5 | 11197174 |  |  |  |  |  |  | -0.19 |  |  |  |  |  |
| BTA-75591-NO-RS | 5 | 12791449 |  |  |  | 7.55 |  |  |  |  |  |  |  |  |
| HAPMAP36616-SCAFFOLD310212_1822 | 5 | 23171537 | -0.55 | -0.14 |  |  |  |  |  |  |  |  |  |  |
| ARS-BFGL-NGS-34254 | 5 | 27287454 | -0.72 |  |  |  |  |  |  |  |  |  |  |  |
| BTA-110099-NO-RS | 5 | 33861705 |  |  |  |  |  |  |  |  | -2.55 |  |  |  |
| ARS-BFGL-NGS-54580 | 5 | 38862744 |  |  | -8.05 |  |  |  |  |  |  |  |  |  |
| BTB-01752230 | 5 | 44578979 |  |  |  |  |  |  | -0.18 |  |  |  |  |  |
| HAPMAP43887-BTA-58386 | 5 | 54045462 |  |  |  |  |  |  |  | 2.16 |  |  |  |  |
| HAPMAP27767-BTA-154179 | 5 | 60263049 |  |  |  |  |  |  | -0.23 |  |  |  |  |  |
| ARS-BFGL-NGS-21052 | 5 | 63365980 |  |  |  |  |  |  |  | 2.18 |  |  |  |  |
| BFGL-NGS-111077 | 5 | 90752558 |  |  |  |  |  |  |  | -1.89 |  |  |  |  |
| HAPMAP48620-BTA-113306 | 5 | 91799509 | 0.68 |  |  |  |  |  |  |  |  |  |  |  |
| ARS-BFGL-NGS-2337 | 5 | 113635679 |  |  |  |  | 0.15 |  |  |  |  |  |  |  |
| ARS-BFGL-NGS-106826 | 5 | 114621971 |  |  |  |  | 0.14 |  |  |  |  |  |  |  |
| BTA-75458-NO-RS | 5 | 120037175 |  |  |  |  |  |  |  |  | 1.94 |  |  |  |
| ARS-BFGL-NGS-17079 | 6 | 13318510 | -0.78 |  |  |  |  |  |  |  |  |  |  |  |
| BTB-00245684 | 6 | 15633977 | -0.65 |  |  |  |  |  |  |  |  |  |  |  |
| BTB-00247622 | 6 | 17522751 |  |  |  |  |  |  |  |  |  |  | 0.83 | 4.96 |
| HAPMAP44568-BTA-77505 | 6 | 17563402 |  |  |  |  |  |  |  |  |  |  |  | -4.88 |
| HAPMAP54751-RS29013947 | 6 | 19307772 |  |  |  |  |  |  |  | -2.36 |  |  |  |  |
| HAPMAP41757-BTA-115774 | 6 | 19918122 |  |  |  |  |  |  |  |  | -1.99 |  |  |  |
| SNP name | Bta | Position (bp) | BF | BIF | HCW | REA | MRB | LM7D | BDFR | BNR | INFR | LNR | SQFR | TLFR |
| ARS-BFGL-NGS-59175 | 6 | 20507429 |  |  |  |  |  |  |  | 2.32 | -1.62 |  |  | -5.83 |
| BTB-01034609 | 6 | 20568920 |  |  | -7.89 |  |  |  |  |  |  |  |  |  |
| BFGL-NGS-114695 | 6 | 21909290 |  |  |  |  | 0.15 |  |  |  |  |  |  |  |
| BTB-01530236 | 6 | 22302502 |  |  |  | 4.76 |  |  |  |  |  |  |  |  |
| ARS-BFGL-NGS-12563 | 6 | 31330398 |  |  | 8.62 |  |  |  |  |  |  |  |  |  |
| HAPMAP59689-RS29020944 | 6 | 36930214 |  |  |  |  |  |  | 0.27 |  |  |  |  |  |
| HAPMAP31922-BTC-034015 | 6 | 37335860 |  |  |  |  |  |  | 0.21 |  |  |  |  |  |
| UA-IFASA-6538 | 6 | 37399296 |  |  | -10.76 |  |  |  |  |  |  |  |  |  |
| HAPMAP26885-BTC-055761 | 6 | 37494040 |  |  | -8.09 |  |  |  |  |  |  |  |  |  |
| HAPMAP25165-BTC-032007 | 6 | 37742740 |  |  | -13.63 |  |  |  |  |  |  |  |  |  |
| HAPMAP25417-BTC-036670 | 6 | 37801349 |  |  | 14.51 |  |  |  |  |  |  |  |  |  |
| HAPMAP26261-BTC-034133 | 6 | 37868743 |  |  | -8.60 |  |  |  |  |  |  |  |  |  |
| BTA-121739-NO-RS | 6 | 38063313 |  |  | -15.19 |  |  |  |  |  |  |  |  |  |
| HAPMAP29922-BTC-033565 | 6 | 38286952 |  |  | 10.54 |  |  |  |  |  |  |  |  |  |
| HAPMAP30134-BTC-034283 | 6 | 38464203 |  |  | -11.37 | -5.48 |  |  |  |  |  |  |  |  |
| HAPMAP26308-BTC-057761 | 6 | 38576012 |  |  | -11.65 |  |  |  |  |  |  |  |  |  |
| ARS-BFGL-NGS-45457 | 6 | 38715250 |  |  | -12.57 | -5.24 |  |  |  |  |  |  |  |  |
| HAPMAP31285-BTC-041097 | 6 | 38869785 |  |  | -10.58 |  |  |  |  |  |  |  |  |  |
| HAPMAP33628-BTC-041023 | 6 | 38939012 |  |  | 12.43 | 4.97 |  |  |  |  |  |  |  |  |
| ARS-BFGL-NGS-2946 | 6 | 39069719 |  |  | 13.60 |  |  |  |  |  |  |  |  |  |
| HAPMAP28546-BTC-072715 | 6 | 39172862 |  |  | -10.44 |  |  |  |  |  |  |  |  |  |
| HAPMAP27537-BTC-060891 | 6 | 39257620 |  |  | -12.46 | -5.77 |  |  |  |  |  |  |  |  |
| HAPMAP31044-BTC-071337 | 6 | 39346170 |  |  | -9.07 |  |  |  |  |  |  |  |  |  |
| BTB-00260450 | 6 | 40063618 |  |  | -11.08 |  |  |  |  |  |  |  |  |  |
| HAPMAP43932-BTA-75850 | 6 | 40086624 |  |  | 9.19 |  |  |  |  |  |  |  |  |  |
| HAPMAP28075-BTC-035688 | 6 | 40377515 |  |  |  |  |  |  | -0.25 |  |  |  |  |  |
| HAPMAP33079-BTA-163567 | 6 | 40629318 |  |  | 10.07 |  |  |  |  |  |  |  |  |  |
| BTA-88763-NO-RS | 6 | 43211180 |  |  |  |  |  |  |  | -2.35 |  |  |  |  |
| BTB-00254199 | 6 | 45017700 |  |  |  |  |  |  | -0.18 |  |  |  |  |  |
| SNP name | Bta | Position (bp) | BF | BIF | HCW | REA | MRB | LM7D | BDFR | BNR | INFR | LNR | SQFR | TLFR |
| HAPMAP34686-BES10_CONTIG763_1215 | 6 | 46867937 |  |  |  |  | -0.17 |  |  |  |  |  |  |  |
| BTB-00255537 | 6 | 48671908 |  |  | 10.27 |  |  |  |  |  |  |  |  |  |
| BTB-01362396 | 6 | 66572393 |  |  |  |  | -0.15 |  |  |  |  |  |  |  |
| ARS-BFGL-NGS-40987 | 6 | 68366910 |  |  |  |  |  |  |  | 2.41 |  |  |  |  |
| HAPMAP42748-BTA-98142 | 6 | 76754229 |  |  |  |  | -0.16 |  |  |  |  |  |  |  |
| BTB-00707438 | 6 | 89962889 |  |  |  | -5.14 |  |  |  |  |  |  |  |  |
| BFGL-NGS-119068 | 6 | 92855916 | -0.59 |  |  |  |  |  |  |  |  |  |  |  |
| HAPMAP39434-BTA-77640 | 6 | 101176530 |  |  |  |  |  | 0.32 |  |  |  |  |  |  |
| HAPMAP23305-BTC-071388 | 6 | 101870226 |  |  |  |  |  | 0.40 |  |  | -1.87 |  |  |  |
| HAPMAP32521-BTC-071427 | 6 | 101886575 |  |  |  |  |  | 0.40 |  |  | -1.91 |  |  |  |
| ARS-BFGL-NGS-26829 | 6 | 106834702 |  |  |  |  |  |  | 0.22 |  |  |  |  |  |
| BFGL-NGS-113112 | 6 | 107214281 | -0.55 |  |  |  |  |  |  |  |  |  |  |  |
| ARS-BFGL-NGS-17313 | 6 | 119216563 |  |  |  |  |  | -0.37 |  |  |  |  |  |  |
| ARS-BFGL-NGS-30829 | 7 | 1293067 |  |  |  |  |  |  | 0.18 |  |  |  |  |  |
| ARS-BFGL-NGS-80352 | 7 | 2916678 |  |  |  |  |  |  |  |  |  | 3.30 |  |  |
| BFGL-NGS-113101 | 7 | 3269338 | -0.51 |  |  |  |  |  |  |  |  |  |  |  |
| HAPMAP48895-BTA-79049 | 7 | 14402009 |  |  |  |  |  | 0.37 |  |  |  |  |  |  |
| ARS-BFGL-NGS-4774 | 7 | 17403976 |  |  |  |  |  |  |  |  |  |  | 0.80 |  |
| BTA-28773-NO-RS | 7 | 19084659 |  | 0.16 |  |  |  |  |  |  |  |  |  |  |
| ARS-BFGL-NGS-26768 | 7 | 32923268 |  |  |  |  |  |  |  |  |  |  |  | -4.67 |
| ARS-BFGL-NGS-96276 | 7 | 33017653 |  |  |  |  |  | 0.36 |  |  |  |  |  |  |
| BFGL-NGS-112944 | 7 | 36599560 |  |  |  |  |  |  |  |  | -1.64 |  |  |  |
| ARS-BFGL-NGS-62351 | 7 | 56471412 |  | 0.16 |  |  |  |  |  |  |  |  |  |  |
| ARS-BFGL-NGS-7098 | 7 | 69867738 |  |  | 9.33 |  |  |  |  |  |  |  |  |  |
| BFGL-NGS-117165 | 7 | 73753347 | 0.51 |  |  |  |  |  |  |  |  |  |  |  |
| HAPMAP27181-BTA-148757 | 7 | 77332002 |  |  |  |  |  |  |  |  |  | -3.83 |  |  |
| BTA-86098-NO-RS | 7 | 88859896 |  |  |  |  |  |  |  |  |  |  | -0.99 |  |
| BTA-119020-NO-RS | 7 | 88971675 |  | -0.16 |  |  |  |  |  |  |  |  | -0.99 |  |
| ARS-BFGL-NGS-43901 | 7 | 98498047 |  | 0.14 |  |  |  |  |  |  |  |  |  |  |
| SNP name | Bta | Position (bp) | BF | BIF | HCW | REA | MRB | LM7D | BDFR | BNR | INFR | LNR | SQFR | TLFR |
| HAPMAP38209-BTA-22552 | 7 | 99845133 |  |  |  |  | -0.18 |  |  |  |  |  |  |  |
| HAPMAP35710-SCAFFOLD255050_10929 | 7 | 108096309 |  |  |  | -5.02 |  |  |  |  |  |  |  |  |
| HAPMAP56111-RS29019494 | 7 | 108638689 |  | 0.17 |  |  |  |  |  |  |  |  |  |  |
| ARS-BFGL-NGS-90219 | 8 | 649400 |  |  |  |  | 0.14 |  |  |  |  |  |  |  |
| ARS-BFGL-NGS-79659 | 8 | 679513 |  |  |  |  | 0.13 |  |  |  |  |  |  |  |
| BTB-01276510 | 8 | 4069868 |  |  |  |  |  |  | -0.28 |  |  |  |  |  |
| BTB-00334763 | 8 | 6342923 |  |  |  |  |  |  | -0.25 |  |  |  |  |  |
| ARS-BFGL-NGS-38391 | 8 | 7495233 |  |  |  |  |  |  | 0.21 |  |  |  |  |  |
| ARS-BFGL-NGS-26236 | 8 | 8539773 |  |  | 10.95 |  |  |  |  |  |  |  |  |  |
| BTA-27910-NO-RS | 8 | 14356874 |  |  |  |  | 0.18 |  |  |  |  |  |  |  |
| ARS-BFGL-NGS-26055 | 8 | 18759713 |  |  |  |  |  |  | -0.18 |  |  |  |  |  |
| BTA-109900-NO-RS | 8 | 22479821 |  |  |  |  |  |  |  |  | 1.51 |  |  |  |
| ARS-BFGL-NGS-40453 | 8 | 34536574 |  |  |  |  |  |  |  |  | 1.48 |  |  |  |
| BTB-01762552 | 8 | 37192348 |  |  |  |  |  |  |  |  |  |  |  | -5.33 |
| HAPMAP49060-BTA-122180 | 8 | 38925412 |  | -0.22 |  | 6.85 |  |  |  |  |  |  |  |  |
| HAPMAP54347-RS29011524 | 8 | 39029952 |  | 0.22 |  | -6.70 |  |  |  |  |  |  |  |  |
| BTA-96984-NO-RS | 8 | 39071586 |  | 0.22 |  | -7.27 |  |  |  |  |  |  |  |  |
| ARS-BFGL-NGS-86183 | 8 | 43497231 |  | -0.13 |  |  |  |  |  |  |  |  |  |  |
| HAPMAP46968-BTA-21559 | 8 | 49524000 | 0.53 |  |  |  |  |  |  |  |  |  |  |  |
| BTB-00348451 | 8 | 52280365 |  |  |  |  |  |  |  |  | -1.47 |  |  |  |
| HAPMAP42531-BTA-38304 | 8 | 53270232 | 0.57 |  |  |  |  |  |  |  |  |  |  |  |
| BTB-00357617 | 8 | 73907982 |  |  |  |  |  |  |  |  | -1.73 |  |  |  |
| ARS-BFGL-NGS-90466 | 8 | 76473229 |  |  |  |  |  | -0.38 |  |  |  |  |  |  |
| ARS-BFGL-NGS-25137 | 8 | 76670107 |  |  |  |  |  | -0.32 |  |  |  |  |  |  |
| HAPMAP60592-RS29025478 | 8 | 79660034 |  |  |  |  |  |  |  |  |  | 3.00 |  |  |
| BFGL-NGS-110505 | 8 | 86613020 |  |  |  |  |  |  |  | 2.26 |  |  |  |  |
| BFGL-NGS-116122 | 8 | 87367415 |  |  |  |  |  |  |  |  |  |  | -0.83 |  |
| HAPMAP41365-BTA-82290 | 8 | 96468448 |  |  |  |  |  |  |  |  |  |  | 0.90 |  |
| ARS-BFGL-NGS-101099 | 8 | 97989837 |  |  |  |  |  |  |  | 1.87 |  |  |  |  |
| SNP name | Bta | Position (bp) | BF | BIF | HCW | REA | MRB | LM7D | BDFR | BNR | INFR | LNR | SQFR | TLFR |
| BTB-01377659 | 8 | 100932178 |  |  |  |  |  |  | -0.19 |  |  |  |  |  |
| HAPMAP27876-BTA-147931 | 8 | 106648392 | 0.88 |  |  |  |  |  |  |  |  |  |  |  |
| BTA-118469-NO-RS | 9 | 1888132 |  |  |  |  |  |  |  |  |  | -2.88 |  |  |
| ARS-BFGL-NGS-74565 | 9 | 14989177 |  |  |  |  |  |  |  |  |  | 3.00 |  |  |
| ARS-BFGL-NGS-105703 | 9 | 15672990 |  |  |  |  |  |  | 0.21 |  |  |  |  |  |
| BTB-01348232 | 9 | 22755204 |  | -0.16 |  |  |  |  |  |  |  |  |  |  |
| BTB-00383772 | 9 | 23538211 |  |  |  |  |  |  | 0.18 |  |  |  |  |  |
| HAPMAP34256-BES9_CONTIG132_328 | 9 | 23855406 |  | 0.13 |  |  |  |  |  |  |  |  |  |  |
| HAPMAP53373-RS29012689 | 9 | 26110323 |  |  |  |  |  |  |  |  | 1.60 |  |  | 4.57 |
| ARS-BFGL-NGS-32323 | 9 | 26785284 |  |  |  |  | 0.16 |  |  |  |  |  |  |  |
| ARS-BFGL-NGS-13783 | 9 | 41072210 |  |  |  |  |  |  |  |  |  |  | 0.78 |  |
| BTA-117313-NO-RS | 9 | 41125489 |  | 0.14 |  |  |  |  |  |  |  |  |  |  |
| HAPMAP25907-BTA-159799 | 9 | 51473366 |  |  |  |  |  | 0.31 |  |  |  |  |  |  |
| BTB-01168267 | 9 | 52221237 |  |  |  |  |  |  |  |  |  |  |  | -4.91 |
| BTB-01176710 | 9 | 52644487 |  |  |  |  | 0.16 |  |  |  | 1.82 | -3.68 |  | 6.33 |
| ARS-BFGL-NGS-12787 | 9 | 52684647 |  |  |  |  | -0.15 |  |  |  |  |  |  |  |
| HAPMAP58670-RS29015048 | 9 | 58624775 |  |  |  |  |  |  |  |  | 1.40 |  |  |  |
| BTB-00394480 | 9 | 59231149 |  |  |  |  |  |  |  | -2.26 |  |  |  |  |
| BTB-00396373 | 9 | 60815205 |  |  |  |  |  |  |  |  | -1.77 |  |  |  |
| HAPMAP44297-BTA-84289 | 9 | 75194809 |  |  |  | -5.48 |  |  |  |  |  |  |  |  |
| BTA-89158-NO-RS | 9 | 79691648 | 0.67 |  |  |  |  |  |  |  |  |  |  |  |
| BTB-01895465 | 9 | 91580703 |  | 0.14 |  |  |  |  |  |  |  |  |  |  |
| ARS-BFGL-NGS-41356 | 9 | 101966211 |  | -0.13 |  |  |  |  |  |  |  |  |  |  |
| ARS-BFGL-NGS-85461 | 9 | 103216021 |  |  |  |  |  |  |  |  |  |  | 0.74 |  |
| ARS-BFGL-NGS-102044 | 9 | 103646025 |  |  |  |  |  |  | -0.20 |  |  |  |  |  |
| BFGL-NGS-115046 | 9 | 104288016 |  | 0.17 |  |  |  |  |  |  |  |  |  |  |
| ARS-BFGL-NGS-102129 | 10 | 1034726 |  |  |  |  |  |  |  |  | -1.57 |  |  |  |
| HAPMAP49460-BTA-114998 | 10 | 2445414 |  |  |  |  |  |  |  |  | 1.61 |  |  |  |
| ARS-BFGL-NGS-40198 | 10 | 2617295 |  |  |  |  |  |  |  |  | 1.83 | -3.14 |  | 5.05 |
| HAPMAP48018-BTA-60331 | 10 | 2729641 |  |  |  |  |  |  |  |  | 1.35 |  |  |  |
| SNP name | Bta | Position (bp) | BF | BIF | HCW | REA | MRB | LM7D | BDFR | BNR | INFR | LNR | SQFR | TLFR |
| BTA-23242-NO-RS | 10 | 6543434 |  |  |  |  |  |  |  | -2.30 |  |  |  |  |
| ARS-BFGL-NGS-106806 | 10 | 9558767 | 0.70 |  |  |  |  |  |  |  |  |  |  |  |
| ARS-BFGL-NGS-39469 | 10 | 28781938 |  |  |  |  |  |  |  |  |  | 3.91 |  |  |
| HAPMAP48024-BTA-62291 | 10 | 37793494 |  |  |  |  |  |  |  |  | -1.54 |  |  |  |
| HAPMAP50767-BTA-72346 | 10 | 60145660 |  |  |  |  |  | 0.41 |  |  |  |  |  |  |
| ARS-BFGL-NGS-31962 | 10 | 89619897 |  |  |  |  |  |  |  |  | -1.54 |  |  |  |
| BTB-00446145 | 10 | 94397322 | 0.57 |  |  |  |  |  |  |  |  |  |  |  |
| HAPMAP44291-BTA-81405 | 10 | 98075367 | 0.77 |  |  |  |  |  |  |  |  |  |  |  |
| ARS-BFGL-NGS-68030 | 11 | 1901787 |  |  |  |  |  |  |  |  |  |  | 0.71 |  |
| HAPMAP54920-RS29026446 | 11 | 1963074 |  | -0.15 |  |  |  |  |  |  |  |  |  |  |
| ARS-BFGL-NGS-31844 | 11 | 9363209 |  |  |  |  |  |  |  |  |  |  |  | -5.19 |
| BTA-92021-NO-RS | 11 | 9385008 |  |  |  |  |  |  |  |  |  |  |  | 4.89 |
| BTB-00458773 | 11 | 10753131 |  |  |  |  |  |  | 0.19 |  |  |  |  |  |
| BFGL-NGS-115446 | 11 | 12267076 |  | -0.15 |  |  |  |  |  |  |  |  |  |  |
| ARS-BFGL-NGS-103158 | 11 | 12363892 |  | 0.14 |  |  |  |  |  |  |  |  |  |  |
| BTB-00463178 | 11 | 16345034 | 0.51 |  |  |  |  |  |  |  |  |  | 0.82 | 4.91 |
| BTB-00463351 | 11 | 16374116 |  |  |  |  |  |  |  |  |  |  | -0.80 | -4.67 |
| BTB-01061117 | 11 | 31495990 |  |  |  |  |  | 0.38 |  |  |  |  |  |  |
| HAPMAP47774-BTA-89811 | 11 | 38512409 |  |  |  |  | 0.16 |  |  |  |  |  |  |  |
| HAPMAP15326-RS29013300 | 11 | 39708839 |  |  |  |  |  |  | -0.18 |  |  |  |  |  |
| ARS-BFGL-NGS-64295 | 11 | 41647520 |  |  |  |  |  |  | -0.21 |  |  |  |  | -5.21 |
| HAPMAP24893-BTA-27904 | 11 | 41740456 |  |  |  |  |  |  | -0.19 |  |  |  |  |  |
| HAPMAP38714-BTA-93410 | 11 | 51392558 |  |  |  | -5.32 |  |  |  |  |  |  |  |  |
| BTA-98135-NO-RS | 11 | 61294221 |  |  |  |  |  |  | 0.22 |  |  |  |  |  |
| BFGL-NGS-110217 | 11 | 62703167 |  |  |  | 6.82 |  |  |  |  |  |  |  |  |
| ARS-BFGL-NGS-52860 | 11 | 65686532 |  |  |  |  | -0.15 |  |  |  |  |  |  |  |
| ARS-BFGL-BAC-15570 | 11 | 69240871 |  |  |  |  |  | -0.44 |  |  |  |  |  |  |
| HAPMAP40478-BTA-106311 | 11 | 77159423 |  |  |  |  |  | 0.44 |  |  |  |  |  |  |
| HAPMAP49851-BTA-107323 | 11 | 79657302 |  |  |  |  |  | 0.32 |  |  |  |  |  |  |
| BFGL-NGS-116520 | 11 | 82182413 |  |  |  |  |  |  |  |  |  |  | -0.98 |  |
| SNP name | Bta | Position (bp) | BF | BIF | HCW | REA | MRB | LM7D | BDFR | BNR | INFR | LNR | SQFR | TLFR |
| ARS-BFGL-NGS-2387 | 11 | 89195128 |  |  |  |  |  |  |  |  |  | -3.34 |  |  |
| HAPMAP31724-BTA-126967 | 11 | 89371911 |  |  |  |  |  |  |  |  | 1.49 |  |  | 4.61 |
| BTA-112448-NO-RS | 11 | 90131383 |  |  |  |  |  |  |  |  |  |  | -0.72 |  |
| ARS-BFGL-NGS-34117 | 11 | 91205321 |  |  |  |  | -0.14 |  |  |  |  |  |  |  |
| BFGL-NGS-113879 | 11 | 96905290 |  |  |  |  |  |  |  | -2.39 |  |  |  |  |
| BFGL-NGS-118549 | 11 | 100221888 |  |  |  |  |  |  |  | 2.24 |  |  |  |  |
| BTB-01103269 | 12 | 2569573 |  |  |  |  | -0.18 |  |  |  |  |  |  |  |
| BTA-31543-NO-RS | 12 | 11752906 |  |  |  |  |  |  |  | 2.24 |  |  |  |  |
| HAPMAP54287-SS46526670 | 12 | 16255818 |  |  |  |  |  |  |  | -2.01 |  |  |  |  |
| BTA-31715-NO-RS | 12 | 16326231 |  |  |  |  |  |  |  | 2.02 |  |  |  |  |
| BTA-115055-NO-RS | 12 | 23774978 |  |  |  |  |  |  |  |  |  |  |  | -6.05 |
| BFGL-NGS-116500 | 12 | 28783549 |  |  |  |  |  |  |  | 2.23 |  |  |  |  |
| HAPMAP52274-RS29017133 | 12 | 43799317 |  |  |  |  |  |  | 0.21 |  |  |  |  |  |
| BTA-11801-NO-RS | 12 | 45268668 |  |  |  |  |  | -0.27 |  |  |  |  |  |  |
| BTB-00490466 | 12 | 47836570 |  |  |  |  |  | 0.40 |  |  |  |  |  |  |
| HAPMAP51019-BTA-65454 | 12 | 56367819 |  |  |  |  |  |  |  | -2.04 |  |  |  |  |
| ARS-BFGL-NGS-96681 | 12 | 56418392 |  |  |  |  |  |  |  | -2.21 |  |  |  |  |
| ARS-BFGL-BAC-14350 | 12 | 59720149 |  |  |  |  |  |  |  | 2.41 |  |  |  |  |
| ARS-BFGL-BAC-14364 | 12 | 67386075 |  |  |  |  |  |  | 0.17 |  |  |  |  |  |
| ARS-BFGL-NGS-75733 | 12 | 77964481 |  |  | -8.26 |  |  |  |  |  |  |  |  |  |
| HAPMAP40904-BTA-120929 | 12 | 80583093 |  |  |  |  |  |  |  |  | 1.83 |  |  |  |
| ARS-BFGL-NGS-108499 | 12 | 84528778 |  |  |  |  |  |  |  |  |  | -3.32 |  |  |
| BTA-31359-NO-RS | 12 | 85500588 |  |  |  |  |  |  |  |  | 1.38 |  |  | 4.46 |
| HAPMAP35553-SCAFFOLD269299_1521 | 12 | 85777352 | -0.57 |  |  |  |  |  |  |  |  |  |  |  |
| BTB-01141508 | 13 | 679009 |  | 0.14 |  |  |  |  |  |  |  |  |  |  |
| ARS-BFGL-NGS-37393 | 13 | 3723531 |  |  |  |  |  |  |  |  |  |  | 0.77 |  |
| BFGL-NGS-116013 | 13 | 4153901 |  |  |  |  |  | 0.51 |  |  |  |  |  |  |
| HAPMAP61019-RS29009751 | 13 | 4693551 |  |  |  |  |  |  |  |  |  |  | 0.82 |  |
| HAPMAP44438-BTA-105375 | 13 | 5992621 |  |  |  |  |  |  |  |  |  |  | -0.78 |  |
| BTA-33932-NO-RS | 13 | 13067288 |  |  |  |  |  |  |  |  |  |  | -0.72 |  |
| SNP name | Bta | Position (bp) | BF | BIF | HCW | REA | MRB | LM7D | BDFR | BNR | INFR | LNR | SQFR | TLFR |
| ARS-BFGL-NGS-109071 | 13 | 14773635 | 0.97 | 0.27 |  |  |  |  |  |  |  |  |  |  |
| BTA-34170-NO-RS | 13 | 15017558 |  |  |  |  |  |  | 0.24 |  |  |  |  |  |
| BFGL-NGS-116613 | 13 | 19227633 |  |  |  |  |  |  |  |  |  |  | -1.10 |  |
| HAPMAP40119-BTA-31847 | 13 | 19773655 |  | 0.15 |  |  |  |  |  |  |  |  |  |  |
| HAPMAP42135-BTA-21541 | 13 | 22445369 |  |  |  |  |  |  | 0.23 |  |  |  |  |  |
| BTA-31894-NO-RS | 13 | 25594852 |  |  |  |  |  |  |  |  |  |  |  | 4.76 |
| HAPMAP59420-SS46527113 | 13 | 35533998 | -0.48 |  |  |  |  |  |  |  |  |  |  |  |
| BTB-01505690 | 13 | 45582166 |  |  |  |  |  | -0.35 |  |  |  |  |  |  |
| ARS-BFGL-BAC-15732 | 13 | 50950127 | -0.54 |  |  |  |  |  |  |  |  |  |  |  |
| ARS-BFGL-NGS-90718 | 13 | 62858269 |  |  | 8.89 |  |  |  |  |  |  |  |  |  |
| ARS-BFGL-NGS-56157 | 13 | 62881877 |  |  | -10.26 |  |  |  |  |  |  |  |  |  |
| BFGL-NGS-110102 | 13 | 66370867 |  |  |  |  | 0.13 |  |  |  |  |  |  |  |
| ARS-BFGL-NGS-109197 | 13 | 66825292 |  |  | 7.92 |  |  |  |  |  |  |  |  |  |
| BFGL-NGS-116471 | 13 | 67178078 |  |  | -7.67 |  |  |  |  |  |  |  |  |  |
| ARS-BFGL-NGS-34968 | 13 | 70262878 | -0.49 |  |  |  |  |  |  |  |  |  |  |  |
| HAPMAP27703-BTC-053907 | 14 | 4583344 |  |  |  |  |  |  |  |  | -1.70 |  |  | -4.72 |
| ARS-BFGL-BAC-24839 | 14 | 5631243 |  |  |  |  |  | -0.35 |  |  |  |  |  |  |
| HAPMAP31968-BTC-056754 | 14 | 6042525 |  |  |  |  |  | 0.30 |  |  |  |  |  |  |
| BFGL-NGS-115374 | 14 | 7104148 |  |  | -10.69 |  |  |  |  |  |  |  |  |  |
| HAPMAP22724-BTC-001541 | 14 | 8730305 | -0.51 |  |  |  |  |  |  |  |  |  |  |  |
| ARS-BFGL-NGS-12878 | 14 | 18670379 |  |  |  |  |  |  |  |  |  |  | 0.73 |  |
| BTB-01532239 | 14 | 24437778 |  |  | 9.26 |  |  |  |  |  |  |  |  |  |
| BFGL-NGS-117404 | 14 | 30498375 |  |  |  | -4.76 |  |  |  |  |  |  |  |  |
| UA-IFASA-7599 | 14 | 35473110 |  |  |  |  |  |  |  |  |  |  |  | -6.10 |
| ARS-BFGL-NGS-75302 | 14 | 38418141 |  |  |  |  |  |  |  |  |  |  | 0.98 |  |
| BTB-01128310 | 14 | 40259420 | -0.71 |  |  |  |  |  |  |  |  |  |  |  |
| ARS-BFGL-NGS-31699 | 14 | 42333424 |  |  |  |  |  | -0.41 |  |  |  |  |  |  |
| ARS-BFGL-NGS-91939 | 14 | 46236763 |  | 0.15 |  |  |  |  |  |  |  |  |  |  |
| BTA-35414-NO-RS | 14 | 71571894 |  |  |  |  |  |  | -0.21 |  |  |  |  |  |
| HAPMAP49562-BTA-29896 | 15 | 9315623 | 0.52 |  |  |  |  |  |  |  |  |  |  |  |
| SNP name | Bta | Position (bp) | BF | BIF | HCW | REA | MRB | LM7D | BDFR | BNR | INFR | LNR | SQFR | TLFR |
| BTA-91820-NO-RS | 15 | 11604685 |  |  | -8.16 |  |  |  |  |  |  |  |  |  |
| BTB-01199261 | 15 | 20435893 |  | 0.13 |  |  |  |  |  |  |  |  |  |  |
| ARS-BFGL-NGS-39079 | 15 | 27654853 |  |  |  |  | 0.16 |  |  |  |  |  |  |  |
| BTA-36962-NO-RS | 15 | 48763311 |  |  |  |  | -0.17 |  |  |  |  |  |  |  |
| ARS-BFGL-NGS-40602 | 15 | 53250782 |  |  | 11.52 | 5.84 |  |  |  |  |  |  |  |  |
| HAPMAP32796-BTA-130010 | 15 | 53372343 |  |  |  | -5.47 |  |  |  |  |  |  |  |  |
| HAPMAP42006-BTA-91398 | 15 | 60904807 | -0.48 |  |  |  |  |  |  |  |  |  |  |  |
| ARS-BFGL-BAC-20016 | 15 | 61044897 |  |  |  | 4.66 |  |  |  |  |  |  |  |  |
| HAPMAP25081-BTA-153963 | 15 | 62057421 |  |  |  |  | 0.20 |  |  |  |  |  |  |  |
| ARS-BFGL-NGS-77090 | 15 | 62557254 |  |  |  | 6.66 |  |  |  |  |  |  |  |  |
| BTA-37461-NO-RS | 15 | 69992123 |  |  | -9.49 |  |  |  |  |  |  |  |  |  |
| ARS-BFGL-NGS-104556 | 15 | 71895324 |  |  |  |  |  |  |  |  |  |  | 0.87 |  |
| BTB-00620646 | 15 | 80069939 |  |  |  |  |  |  |  | -2.14 |  |  |  |  |
| ARS-BFGL-NGS-68395 | 16 | 30626532 |  |  |  |  |  |  |  |  |  |  | -0.86 |  |
| ARS-BFGL-NGS-4710 | 16 | 34346915 |  | 0.18 |  |  |  |  |  |  |  |  |  |  |
| HAPMAP46217-BTA-38576 | 16 | 42730174 |  | -0.13 |  |  |  |  |  |  |  |  |  |  |
| UA-IFASA-6048 | 16 | 42806930 |  | 0.13 |  |  |  |  |  |  |  |  |  |  |
| ARS-BFGL-NGS-17391 | 16 | 51833247 |  | 0.20 |  | -6.13 |  |  |  |  |  |  |  |  |
| ARS-BFGL-NGS-43477 | 16 | 53554791 |  |  | -10.09 |  |  |  |  |  |  |  |  |  |
| ARS-BFGL-NGS-29043 | 16 | 54691747 |  |  |  |  |  |  |  | 1.83 |  |  |  |  |
| ARS-BFGL-NGS-20204 | 16 | 71163115 |  |  |  |  |  |  |  | 2.04 |  |  |  |  |
| ARS-BFGL-NGS-43704 | 16 | 71349209 |  |  |  |  |  |  |  |  | 1.52 |  |  |  |
| HAPMAP47281-BTA-40051 | 16 | 72931875 |  |  |  |  |  |  |  | -2.13 |  |  |  |  |
| ARS-BFGL-NGS-103526 | 16 | 77286529 | -0.89 |  |  |  |  |  |  |  |  |  |  |  |
| HAPMAP41531-BTA-40863 | 17 | 446792 |  |  |  |  |  |  |  |  | -1.56 |  |  |  |
| HAPMAP36186-SCAFFOLD206949_1268 | 17 | 6556513 |  |  |  | 4.73 |  |  |  |  |  |  |  |  |
| HAPMAP34622-BES10_CONTIG518_1595 | 17 | 7472614 |  |  |  |  |  |  |  |  |  | -3.27 |  |  |
| BTA-19257-NO-RS | 17 | 14020692 |  |  |  |  |  |  |  |  |  |  | -1.18 |  |
| ARS-BFGL-NGS-104805 | 17 | 14044442 |  |  |  |  |  |  |  |  |  |  | -1.18 |  |
| SNP name | Bta | Position (bp) | BF | BIF | HCW | REA | MRB | LM7D | BDFR | BNR | INFR | LNR | SQFR | TLFR |
| BTB-01297598 | 17 | 14080531 |  |  |  |  |  |  |  |  |  |  | -1.21 |  |
| BTB-00674231 | 17 | 14814012 |  | -0.17 |  |  |  |  |  |  |  |  |  |  |
| BTB-00676077 | 17 | 29057538 |  |  |  |  |  | -0.30 |  |  |  |  |  |  |
| BTB-00116464 | 17 | 45258744 |  |  |  |  | 0.15 |  |  |  |  |  |  |  |
| ARS-BFGL-NGS-94070 | 17 | 53697142 |  |  |  | -6.90 |  |  |  |  |  |  |  |  |
| HAPMAP53501-RS29016784 | 17 | 64416614 |  |  |  |  |  |  |  |  | 1.56 |  |  |  |
| ARS-BFGL-NGS-104905 | 17 | 65425899 |  |  |  |  |  |  | 0.21 |  |  |  |  |  |
| BTB-00698623 | 18 | 8805310 |  |  |  |  |  |  |  |  |  | 4.23 |  |  |
| ARS-BFGL-NGS-98352 | 18 | 19553485 |  |  | -9.59 |  |  |  |  |  |  |  |  |  |
| BFGL-NGS-118855 | 18 | 26527686 |  |  |  |  |  |  |  |  |  | -3.11 | 0.84 | 5.08 |
| BTB-00710625 | 18 | 40464954 |  |  |  |  | 0.16 |  |  |  |  |  |  |  |
| ARS-BFGL-NGS-26944 | 18 | 41595185 |  |  |  |  |  |  |  |  | -1.76 |  |  |  |
| HAPMAP52325-RS29020544 | 18 | 43327273 |  | 0.14 |  | -5.16 |  |  |  |  | 1.60 |  |  |  |
| BFGL-NGS-119814 | 18 | 48747276 |  |  |  |  |  |  | -0.24 |  |  |  |  |  |
| ARS-BFGL-NGS-42247 | 18 | 50748739 |  | -0.13 |  |  |  |  |  |  |  |  |  |  |
| ARS-BFGL-NGS-34874 | 18 | 51896291 |  |  |  | -4.86 |  |  |  |  |  |  |  |  |
| HAPMAP42167-BTA-28651 | 18 | 51917582 |  |  |  | -5.46 |  |  |  |  |  |  |  |  |
| HAPMAP44043-BTA-24219 | 18 | 60205583 |  |  |  |  |  |  | 0.26 |  |  |  |  |  |
| HAPMAP32869-BTA-161633 | 18 | 65174876 |  |  |  |  | 0.18 |  |  |  |  |  |  |  |
| ARS-BFGL-NGS-25347 | 19 | 7089496 |  |  |  |  |  |  |  | 2.21 |  |  |  |  |
| ARS-BFGL-NGS-37177 | 19 | 13154786 |  |  |  |  |  | -0.43 |  |  |  |  |  |  |
| ARS-BFGL-NGS-103553 | 19 | 28595703 |  |  |  |  |  |  |  |  |  |  | -0.85 |  |
| HAPMAP54918-RS29023719 | 19 | 28677989 |  |  |  |  |  |  |  |  |  |  | -0.85 |  |
| ARS-BFGL-NGS-4573 | 19 | 31113707 |  |  |  |  |  |  |  |  |  |  | -0.74 |  |
| BTA-123276-NO-RS | 19 | 41950232 |  |  |  |  |  | -0.34 |  |  |  |  |  |  |
| ARS-BFGL-BAC-35052 | 19 | 48280175 |  |  |  |  |  | 0.45 |  |  |  |  |  |  |
| UA-IFASA-7593 | 19 | 49375655 |  |  |  |  |  |  |  |  | -1.73 |  |  |  |
| HAPMAP43270-BTA-45709 | 19 | 49473960 |  |  |  |  |  |  |  |  | 1.73 |  |  |  |
| ARS-BFGL-NGS-86346 | 19 | 53871632 |  |  |  |  |  | -0.49 |  |  |  |  |  |  |
| ARS-BFGL-NGS-88651 | 19 | 55235501 |  |  |  |  |  | -0.30 |  |  |  |  |  |  |
| SNP name | Bta | Position (bp) | BF | BIF | HCW | REA | MRB | LM7D | BDFR | BNR | INFR | LNR | SQFR | TLFR |
| ARS-BFGL-NGS-43623 | 19 | 55257191 |  |  |  |  |  | 0.29 |  |  |  |  |  |  |
| HAPMAP49184-BTA-45954 | 19 | 55295443 |  | -0.13 |  |  |  |  |  |  |  |  |  |  |
| ARS-BFGL-NGS-71489 | 19 | 57098859 |  |  |  |  | 0.15 |  |  |  |  |  |  |  |
| HAPMAP51158-BTA-117076 | 20 | 199074 |  |  | -8.40 |  |  |  |  |  |  |  |  |  |
| ARS-BFGL-NGS-65452 | 20 | 593947 |  |  |  |  |  |  |  |  | 2.20 |  |  | 7.65 |
| HAPMAP54098-RS29010434 | 20 | 4395656 |  |  |  |  |  |  |  | -2.49 |  |  |  |  |
| ARS-BFGL-NGS-28479 | 20 | 4425557 | -0.45 |  |  |  |  |  |  |  |  |  |  |  |
| BTB-00144202 | 20 | 4713452 |  |  |  |  |  |  |  | -2.41 |  |  |  |  |
| BTB-00144185 | 20 | 4746836 |  |  |  |  |  |  |  | -2.15 |  |  |  |  |
| HAPMAP48851-BTA-68711 | 20 | 4788269 |  |  |  |  |  |  |  | -2.23 |  |  |  |  |
| HAPMAP53870-RS29020081 | 20 | 5987334 |  |  |  |  |  |  |  |  | 1.79 |  |  | 5.25 |
| ARS-BFGL-NGS-78095 | 20 | 21073349 |  |  |  |  |  |  |  |  |  | -3.02 |  |  |
| BFGL-NGS-113813 | 20 | 21343153 |  |  |  |  |  |  |  |  |  |  |  | 6.34 |
| ARS-BFGL-NGS-20706 | 20 | 21674110 |  |  |  |  |  |  |  | 1.96 |  |  |  |  |
| HAPMAP51840-BTA-50193 | 20 | 27997551 |  |  |  | -7.01 |  |  |  |  |  |  |  |  |
| ARS-BFGL-NGS-70916 | 20 | 28538251 |  |  |  |  |  |  |  | -1.89 |  |  |  |  |
| HAPMAP54258-RS29018641 | 20 | 31151035 |  |  |  |  |  |  |  |  |  | 3.57 |  |  |
| INRA-620 | 20 | 37440474 |  |  |  |  |  | -0.38 |  |  |  |  |  |  |
| BTB-00784470 | 20 | 43381899 |  |  |  |  |  |  |  | 2.17 |  |  |  |  |
| BTB-02019272 | 20 | 43418343 |  |  |  |  |  |  |  | 2.13 |  |  |  |  |
| HAPMAP26424-BTA-149429 | 20 | 43453092 |  |  |  |  |  |  |  | 2.17 |  |  |  |  |
| HAPMAP51988-BTA-92052 | 20 | 43489592 |  |  |  |  |  |  |  | 2.19 |  |  |  |  |
| BTA-92055-NO-RS | 20 | 43612963 |  |  |  |  |  |  | -0.27 |  |  |  |  |  |
| ARS-BFGL-NGS-79994 | 20 | 51732785 | 0.55 |  |  |  |  |  |  |  |  |  |  |  |
| BTB-01583562 | 20 | 55425112 |  |  |  |  |  | 0.38 |  |  |  |  |  |  |
| HAPMAP51375-BTA-105537 | 20 | 60783431 | 0.53 |  |  |  |  |  |  |  |  |  |  |  |
| ARS-BFGL-NGS-14979 | 20 | 61016074 |  | 0.16 |  |  |  |  |  |  |  |  |  |  |
| BTB-00793885 | 20 | 61311513 |  |  | 8.40 |  |  |  |  |  |  |  |  |  |
| HAPMAP40754-BTA-50982 | 20 | 61863283 |  |  |  |  |  |  | -0.24 |  |  |  |  |  |
| ARS-BFGL-NGS-69670 | 20 | 64185456 |  |  |  |  |  |  |  | -2.58 |  |  |  |  |
| SNP name | Bta | Position (bp) | BF | BIF | HCW | REA | MRB | LM7D | BDFR | BNR | INFR | LNR | SQFR | TLFR |
| HAPMAP43283-BTA-51056 | 20 | 64648481 |  |  |  |  |  |  |  | -2.08 |  |  |  | 4.36 |
| ARS-BFGL-NGS-77469 | 20 | 68570594 |  |  |  |  |  |  |  |  |  |  |  | -4.33 |
| HAPMAP51246-BTA-51263 | 20 | 69504928 |  |  |  |  |  |  | -0.19 |  |  |  |  |  |
| BTB-01714719 | 21 | 10042947 |  |  |  |  |  |  | -0.17 |  |  |  |  |  |
| HAPMAP40438-BTA-51877 | 21 | 24805298 | 0.51 |  |  |  |  |  |  |  |  |  |  |  |
| ARS-BFGL-NGS-103205 | 21 | 27166480 |  |  |  |  |  | 0.37 |  |  |  |  |  |  |
| HAPMAP51843-BTA-52149 | 21 | 36228253 | -0.60 |  |  |  |  |  |  |  |  |  |  |  |
| BTB-01533089 | 21 | 39716280 |  |  | -11.19 |  |  |  |  |  |  |  |  |  |
| ARS-BFGL-BAC-29181 | 21 | 42357276 |  |  |  | -4.49 |  |  |  |  |  |  |  |  |
| HAPMAP44324-BTA-97029 | 21 | 43304015 |  |  |  | 5.07 |  |  |  |  |  |  |  |  |
| BFGL-NGS-116131 | 21 | 43326877 |  |  |  | 4.99 |  |  |  |  |  |  |  |  |
| HAPMAP39755-BTA-52639 | 21 | 55574197 |  |  |  |  | 0.16 |  |  |  |  |  |  |  |
| ARS-BFGL-NGS-24785 | 21 | 58060052 |  | -0.19 |  |  |  |  |  |  |  |  |  | -6.92 |
| ARS-BFGL-NGS-41245 | 21 | 58188882 |  | 0.23 |  |  |  |  |  |  |  |  |  |  |
| BFGL-NGS-118390 | 21 | 64129449 |  |  |  |  |  |  |  |  |  | -4.41 |  |  |
| BFGL-NGS-119656 | 21 | 67963614 |  |  |  | 6.42 |  |  |  |  |  |  |  |  |
| BFGL-NGS-112796 | 22 | 2857608 |  |  |  |  | -0.14 |  |  |  |  |  |  |  |
| ARS-BFGL-NGS-21706 | 22 | 13798228 |  |  |  |  |  |  |  | -1.88 |  |  |  |  |
| BTA-117276-NO-RS | 22 | 19930886 |  |  |  | 5.06 |  |  |  |  |  |  |  |  |
| BTA-54060-NO-RS | 22 | 27427804 |  |  |  | 4.86 |  |  |  |  |  |  |  |  |
| ARS-BFGL-NGS-68862 | 22 | 32208759 |  |  |  |  |  |  |  |  | -1.44 |  |  |  |
| BFGL-NGS-119427 | 22 | 37455074 |  | 0.14 |  |  |  |  |  |  |  |  |  |  |
| BTA-54278-NO-RS | 22 | 38341013 |  |  |  |  |  |  |  |  | -1.85 |  |  |  |
| ARS-BFGL-NGS-4194 | 22 | 40997991 |  |  |  |  |  |  |  | -2.00 |  |  |  |  |
| HAPMAP43133-BTA-106470 | 22 | 41974372 | 0.51 |  |  |  |  |  |  |  |  |  |  |  |
| ARS-BFGL-NGS-20777 | 22 | 42202220 | 0.56 |  |  |  |  |  |  |  |  |  |  |  |
| BFGL-NGS-112869 | 22 | 44619321 | -0.59 |  |  |  |  |  |  | 2.38 |  |  |  |  |
| ARS-BFGL-NGS-5170 | 22 | 47762087 |  |  | -9.44 | -5.32 |  |  |  |  |  |  |  |  |
| ARS-BFGL-NGS-18542 | 22 | 47827050 |  |  | -8.57 |  |  |  |  |  |  |  |  |  |
| BTB-00850807 | 22 | 48063014 |  |  |  |  |  | 0.32 |  |  |  |  |  |  |
| SNP name | Bta | Position (bp) | BF | BIF | HCW | REA | MRB | LM7D | BDFR | BNR | INFR | LNR | SQFR | TLFR |
| BTA-54761-NO-RS | 22 | 50926015 |  | -0.14 |  |  |  |  |  |  |  |  |  |  |
| BFGL-NGS-110743 | 22 | 53377535 |  |  |  |  | 0.17 |  |  |  |  |  |  |  |
| ARS-BFGL-NGS-106287 | 22 | 54645178 |  |  |  |  |  | -0.40 |  |  |  |  |  |  |
| HAPMAP60475-RS29022896 | 23 | 7191371 |  |  |  | -4.75 |  |  |  |  |  |  |  |  |
| BFGL-NGS-111195 | 23 | 15375447 |  |  |  |  |  |  |  |  |  |  |  | 4.41 |
| ARS-BFGL-NGS-34057 | 23 | 15907479 |  |  | -8.76 |  |  |  |  |  |  |  |  |  |
| HAPMAP57208-RS29025986 | 23 | 16601543 |  |  | 8.67 |  |  |  |  |  |  |  |  |  |
| ARS-BFGL-NGS-109142 | 23 | 25552635 |  |  |  |  |  |  |  |  |  |  | -0.85 |  |
| BFGL-NGS-110774 | 23 | 29150525 |  | 0.13 |  |  |  |  |  |  |  |  |  |  |
| HAPMAP52976-RS29018100 | 23 | 35970763 |  |  |  |  |  |  |  |  | 1.57 |  |  |  |
| ARS-BFGL-NGS-62081 | 23 | 37292633 | -0.71 |  |  |  |  |  |  |  |  |  | -0.89 |  |
| BFGL-NGS-115866 | 23 | 38224271 |  |  |  |  |  |  |  |  |  |  | -0.86 |  |
| BTB-00577027 | 23 | 42384884 |  |  |  |  |  |  | -0.20 |  |  |  |  |  |
| HAPMAP54312-RS29012588 | 23 | 43789029 |  |  | -7.61 |  |  |  |  |  |  |  |  |  |
| ARS-BFGL-NGS-103469 | 24 | 632760 |  |  | 8.50 |  |  |  |  |  |  |  |  |  |
| BFGL-NGS-111867 | 24 | 858718 | -0.51 |  |  |  |  |  |  |  |  |  |  |  |
| HAPMAP41588-BTA-57926 | 24 | 2936114 | 0.56 |  |  |  |  |  |  |  |  |  |  |  |
| BTA-58999-NO-RS | 24 | 8312970 |  |  |  | -5.47 |  |  |  |  |  |  |  |  |
| ARS-BFGL-NGS-31355 | 24 | 11888700 |  |  |  |  |  |  |  |  |  |  |  | 5.30 |
| BTA-57636-NO-RS | 24 | 22403126 |  |  |  |  |  |  | 0.21 |  |  |  |  |  |
| HAPMAP47332-BTA-58247 | 24 | 45052793 |  |  |  |  |  |  |  | -2.76 |  |  |  |  |
| ARS-BFGL-NGS-96714 | 24 | 51254221 |  |  |  |  |  | 0.40 |  |  |  |  |  |  |
| ARS-BFGL-NGS-62237 | 25 | 813455 |  |  |  |  |  |  | 0.18 |  |  |  |  |  |
| BFGL-NGS-111712 | 25 | 1160378 |  | 0.14 |  |  |  |  |  |  |  |  |  |  |
| HAPMAP29768-BTC-016149 | 25 | 1205232 |  | 0.13 |  |  |  |  |  |  |  |  |  |  |
| HAPMAP32426-BTC-000132 | 25 | 5759625 |  |  |  |  |  |  |  | -1.90 |  |  |  |  |
| ARS-BFGL-NGS-68584 | 25 | 8051126 |  |  |  |  |  |  |  |  |  |  | 0.71 |  |
| ARS-BFGL-NGS-9967 | 25 | 11595587 |  |  |  |  |  |  |  |  |  |  |  | 4.62 |
| ARS-BFGL-NGS-6013 | 25 | 14110867 |  |  | 8.98 |  |  |  |  |  |  |  |  |  |
| ARS-BFGL-NGS-26313 | 25 | 15006757 |  |  |  |  |  |  |  |  | -1.31 |  |  |  |
| SNP name | Bta | Position (bp) | BF | BIF | HCW | REA | MRB | LM7D | BDFR | BNR | INFR | LNR | SQFR | TLFR |
| ARS-BFGL-NGS-74312 | 25 | 16396397 |  |  |  |  |  |  |  |  | -1.52 |  |  |  |
| ARS-BFGL-NGS-75298 | 25 | 28272468 |  | 0.16 |  |  |  |  |  |  |  |  |  |  |
| ARS-BFGL-BAC-46980 | 25 | 33603349 |  |  |  |  |  |  |  |  | -1.39 |  |  |  |
| HAPMAP48825-BTA-60019 | 25 | 33920843 |  |  |  |  |  | -0.32 |  |  |  |  |  |  |
| ARS-BFGL-NGS-52638 | 25 | 35801651 |  |  |  |  |  |  |  |  | 1.48 |  |  |  |
| HAPMAP58114-RS29014516 | 25 | 39312988 |  |  |  |  |  |  | -0.19 |  |  |  |  |  |
| ARS-BFGL-NGS-3041 | 25 | 39341426 |  |  |  |  |  |  | -0.20 |  |  |  |  |  |
| BTA-113604-NO-RS | 26 | 8356096 |  | -0.22 |  | 8.70 |  |  |  |  |  |  |  |  |
| ARS-BFGL-NGS-25406 | 26 | 14869035 |  |  |  |  |  |  |  | -2.90 |  |  |  |  |
| HAPMAP51474-BTA-62082 | 26 | 17360205 |  |  |  |  | -0.18 |  |  |  |  |  |  |  |
| UA-IFASA-9551 | 26 | 17389674 | -0.73 |  |  |  |  |  |  |  |  |  |  |  |
| BTB-01985838 | 26 | 19973761 |  |  |  |  |  |  | 0.17 |  |  |  |  |  |
| ARS-BFGL-NGS-5577 | 26 | 22215515 |  |  |  | -5.00 |  |  |  |  |  |  |  |  |
| ARS-BFGL-NGS-36064 | 26 | 22257931 |  |  |  | -4.96 |  |  |  |  |  |  |  |  |
| BTA-91041-NO-RS | 26 | 32792279 |  | -0.13 |  |  |  |  |  |  |  |  |  |  |
| HAPMAP31761-BTA-138979 | 26 | 38726082 |  |  |  |  |  |  | 0.33 |  |  |  |  |  |
| ARS-BFGL-NGS-33804 | 26 | 41709612 |  |  |  |  |  |  |  | 2.49 |  |  |  |  |
| ARS-BFGL-NGS-108850 | 26 | 42896381 | -0.86 |  |  |  |  |  |  |  |  |  |  |  |
| ARS-BFGL-NGS-15401 | 26 | 43801835 |  |  |  |  |  |  | -0.18 |  |  |  |  |  |
| ARS-BFGL-NGS-54155 | 27 | 6901933 |  |  |  | 4.79 |  |  |  |  |  |  |  |  |
| ARS-BFGL-NGS-102850 | 27 | 14931898 |  |  |  |  |  |  |  |  | 1.75 |  |  | 5.61 |
| ARS-BFGL-NGS-100615 | 27 | 20863121 |  |  |  |  |  |  | -0.23 |  |  |  |  |  |
| BFGL-NGS-112500 | 27 | 23463596 |  |  |  |  |  |  |  |  |  |  | -0.79 |  |
| BTA-62498-NO-RS | 27 | 24386563 | 0.77 |  |  |  |  |  |  |  |  |  |  |  |
| ARS-BFGL-NGS-43776 | 27 | 24576801 |  |  |  |  |  |  | 0.21 |  |  |  |  |  |
| BTB-00964012 | 27 | 24859486 | -0.71 | -0.19 |  |  |  |  |  |  |  |  | -1.13 | -6.16 |
| BTB-00964618 | 27 | 25529207 | -0.50 |  |  |  |  |  |  |  |  |  |  |  |
| BTA-38129-NO-RS | 27 | 29893059 |  |  |  | 6.95 |  |  |  |  |  |  |  |  |
| ARS-BFGL-NGS-92338 | 27 | 37120275 | -0.66 |  |  |  |  |  |  |  |  |  |  |  |
| HAPMAP50424-BTA-63130 | 27 | 41726707 |  |  |  |  |  | 0.36 |  |  |  |  |  |  |
| SNP name | Bta | Position (bp) | BF | BIF | HCW | REA | MRB | LM7D | BDFR | BNR | INFR | LNR | SQFR | TLFR |
| HAPMAP34350-BES8_CONTIG562_678 | 28 | 11932557 |  |  |  |  |  | 0.43 |  |  |  |  |  |  |
| ARS-BFGL-NGS-32797 | 28 | 13589307 |  |  |  |  |  |  |  |  |  |  |  | 5.56 |
| HAPMAP48306-BTA-36540 | 28 | 38449335 |  |  |  |  | -0.16 |  |  |  |  |  |  |  |
| ARS-BFGL-NGS-34173 | 29 | 22898633 |  |  |  |  |  | 0.35 |  |  |  |  |  |  |
| HAPMAP48311-BTA-38136 | 29 | 23262727 |  |  |  |  |  |  | 0.25 |  |  |  |  |  |
| ARS-BFGL-NGS-91937 | 29 | 25587657 |  |  |  |  | -0.14 |  |  |  |  |  |  |  |
| BFGL-NGS-114570 | 29 | 25759091 |  |  |  |  |  | -0.33 |  |  |  |  |  |  |
| ARS-BFGL-NGS-29332 | 29 | 25780595 |  |  |  |  |  | 0.35 |  |  |  |  |  |  |
| ARS-BFGL-NGS-86495 | 29 | 40695267 |  |  |  |  |  | -0.31 |  |  |  |  |  |  |
| BFGL-NGS-110940 | 29 | 43914923 |  |  |  |  |  | 0.45 |  |  |  |  |  |  |
| CAPN1_1 | 29 | 44069063 |  |  |  |  |  | 0.50 |  |  |  |  |  |  |
| ARS-BFGL-NGS-35836 | 29 | 48644167 |  |  |  |  | 0.15 |  |  |  |  |  |  |  |
| ARS-BFGL-NGS-56596 | 29 | 48843374 |  |  |  |  |  |  |  | 2.73 |  |  |  |  |
| HAPMAP57188-RS29027555 | 29 | 49867074 |  |  | -8.54 |  |  |  |  |  |  |  |  |  |
| ARS-BFGL-NGS-109317 | 29 | 49906123 |  |  | -10.43 |  |  |  |  |  |  |  |  |  |
